# Characterization and clinical management of adverse events following treatment with repotrectinib: a TRIDENT-1 analysis

**DOI:** 10.1093/oncolo/oyag137

**Published:** 2026-04-16

**Authors:** Alexander Drilon, Byoung Chul Cho, D Ross Camidge, Misako Nagasaka, Benjamin Besse, Benjamin Solomon, Koichi Goto, Jürgen Wolf, Sanjay Popat, Enriqueta Felip, Nong Yang, Adrianus Johannes de Langen, Shun Lu, Vamsidhar Velcheti, Andrew L Lin, Christophe Y Calvet, Li Li, Marina Tschaika, Salman Afsar, Haisu Yang, Jessica J Lin

**Affiliations:** Memorial Sloan Kettering Cancer Center, Weill Cornell Medical College, New York, NY, United States; Yonsei Cancer Center, Yonsei University College of Medicine, Seoul, Republic of Korea; Anschutz Medical Campus, University of Colorado, Aurora, CO, United States; School of Medicine, University of California, Irvine, Orange, CA, United States; Gustave Roussy Cancer Center, Paris-Saclay University, Villejuif, France; Peter MacCallum Cancer Centre, Melbourne, Australia; National Cancer Center Hospital East, Kashiwa, Japan; Centrum für Integrierte Onkologie—Uniklinik Köln, Köln, Germany; The Royal Marsden NHS Foundation Trust & The Institute of Cancer Research, London, United Kingdom; Vall d’Hebron University Hospital, Vall d’Hebron Institute of Oncology, Barcelona, Spain; The Second People’s Hospital of Hunan Province, Hunan, China; Netherlands Cancer Institute, Amsterdam, The Netherlands; Department of Oncology, Shanghai Chest Hospital, Shanghai, China; Mayo Clinic, Jacksonville, FL,United States; Memorial Sloan Kettering Cancer Center, Weill Cornell Medical College, New York, NY, United States; Bristol Myers Squibb, Princeton, NJ, United States; Bristol Myers Squibb, Princeton, NJ, United States; Bristol Myers Squibb, Princeton, NJ, United States; Bristol Myers Squibb, Princeton, NJ, United States; Bristol Myers Squibb, Princeton, NJ, United States; Harvard Medical School, Mass General Brigham Cancer Institute, Boston, MA, United States

**Keywords:** Repotrectinib, TRIDENT-1, safety management, NSCLC, clinical management

## Abstract

**Background:**

Repotrectinib, a next-generation ROS1/TRK tyrosine kinase inhibitor, is approved for *ROS1* fusion-positive non-small cell lung cancer and *NTRK* fusion-positive solid tumors. Its side effects and safety management strategies require further characterization.

**Patients and Methods:**

The safety profile of repotrectinib (treatment-emergent/related adverse events [TEAEs/TRAEs]) was established in patients who initiated treatment at the recommended dose (160 mg daily [QD] for 14 days, then 160 mg twice daily [BID]) across all cohorts of the global, multicenter phase 1/2 TRIDENT-1 study. AE management strategies were outlined.

**Results:**

In 472 patients, the most common TRAEs (dizziness [58%] and dysgeusia [50%]) were likely TRK inhibition-related. Median relative dose intensity was 90%; 14% (*n* = 66/472) of patients did not increase their initial QD dose to BID (mostly due to CNS AEs). Rates of dizziness (median onset, 7 days) were similar in patients with/without baseline brain metastases. Dose modifications downgraded severity or resolved dizziness in 78% of patients; 58% of patients had pharmacologic intervention without dose modification. Dizziness was downgraded/resolved in 62% (*n* = 120/195) of patients who did not receive dose modification or pharmacologic intervention. Treatment-related cognitive impairment and weight gain occurred in 19% and 12% of patients, respectively. Treatment-emergent withdrawal pain occurred in 14% of patients (median resolution time, 2.1 weeks). Dose interruption and reduction from TRAEs occurred in 39% and 38% of patients, respectively; 10% reported later re-escalation back to 160 mg BID.

**Conclusion:**

Many repotrectinib AEs, including neurological AEs secondary to TRK inhibition, were mitigated with appropriate management, including dose modification and/or pharmacologic intervention.

Implications for Practice Repotrectinib is a next-generation ROS1 and TRK tyrosine kinase inhibitor with clinically meaningful efficacy in the treatment of patients with advanced *ROS1*+ non-small cell lung cancer and *NTRK*+ solid tumors in the TRIDENT-1 trial. This article summarizes and provides guidance on the management of repotrectinib adverse events (AEs). Proper management can help mitigate AEs, including AEs mediated by TRK inhibition. Dose modification and pharmacologic intervention are effective treatment strategies.

## Introduction

*ROS1* fusions occur in up to 2% of patients with non-small cell lung cancer (NSCLC).^1^  *NTRK1*, *NTRK2*, and *NTRK3* fusions are found in various tumor types and occur in up to 1% of all solid tumors.^2^^,^^3^  *ROS1* is a proto-oncogene that encodes a receptor tyrosine kinase whose physiological role is unknown.^4^  *NTRK* genes are predominantly expressed in neuronal tissue and play an essential role during embryonic development and in the normal function of the nervous system.^3^ The *NTRK* gene products, the TRK family of receptors (TRKA, TRKB, TRKC), are important in neuronal development and differentiation, including survival, proliferation, and differentiation of neurons, synapse formation and plasticity, membrane trafficking, and axon and dendrite growth; therefore, TRK inhibition is known to cause neurologic toxicities.^2^^,^^5^

Early generation ROS1 (crizotinib and entrectinib) and TRK (larotrectinib and entrectinib) tyrosine kinase inhibitors (TKIs) are active in patients with advanced *ROS1* fusion-positive (*ROS1*+) NSCLC and *NTRK*1/2/3 fusion-positive (*NTRK*+) solid tumors, respectively. However, resistance ultimately emerges and leads to disease progression.^6–9^ This clinical limitation underscores the need for additional therapeutic options that overcome or prevent resistance and promote durable responses.

Repotrectinib is a next-generation ROS1 and TRK TKI with a compact macrocyclic structure that binds completely inside the ATP binding pocket (circumventing steric interference from potential resistance mutations) and displays substantial intracranial activity.^10^ Results from the phase 1/2 TRIDENT-1 trial led to approval of repotrectinib in multiple countries for the treatment of adult patients with locally advanced/metastatic *ROS1*+ NSCLC and for adult or pediatric patients with *NTRK*+ locally advanced/metastatic solid tumors.^11–16^ The recommended repotrectinib dosage is 160 mg taken orally once daily (QD) with or without food for 14 days, then 160 mg twice daily (BID) until disease progression or unacceptable toxicity.^11^

Although initial safety analyses of repotrectinib were previously reported and prescribing information of repotrectinib includes recommendations for dose modifications to manage adverse events (AEs),^11^^,^^12^ further characterization of repotrectinib’s safety profile and guidance on AE management are critical to inform clinical practice. Here, we present a comprehensive safety analysis in all patients treated in TRIDENT-1 at the recommended dose, including a larger population with a longer follow-up compared to the previous report.^12^ We also report clinical management of select clinically relevant AEs, including central nervous system (CNS) AEs (dizziness, dysgeusia, cognitive impairment, and withdrawal pain), pneumonitis/interstitial lung disease (ILD), myalgia/increased creatine phosphokinase (CPK), weight increase, fractures, edema, cardiac AEs, hepatotoxicity, and vision disorders.

## Materials and methods

### Trial design and treatment

TRIDENT-1 (NCT03093116) is an ongoing phase 1/2, open-label, multicenter study of repotrectinib in patients with *ROS1*, *NTRK*, or *ALK* fusion–positive locally advanced/metastatic solid tumors. Patients in phase 2 were assigned to 1 of 6 expansion cohorts based on gene fusion and prior treatment (Figure S1). Eligibility criteria, trial endpoints, and dose escalation criteria in phase 2 were previously reported.^12^

Based on TRIDENT-1 phase 1 results, the recommended dose of repotrectinib was 160 mg QD for 14 days, followed by 160 mg BID; the rationale for this dosing was previously published.^11^^,^^12^ Recommended dose modification guidelines permitted dose interruptions as needed until toxicity resolution and/or dose reduction by up to 2 dose levels (160 mg QD to 120 mg QD to 80 mg QD; 160 mg BID to 120 mg BID to 80 mg BID) based on the type of toxicity, severity, persistence, and recurrence of the event (Table S1). The steady state repotrectinib terminal half-life is approximately 40.3 hours for patients with cancer.^11^

### Safety analysis

Types of AEs and their incidence, severity (graded per the National Cancer Institute Common Terminology Criteria for Adverse Events [CTCAE], version 4.03), time to onset, time to resolution (for event of longest duration), seriousness, dose modifications, treatment discontinuations, and relatedness to repotrectinib treatment were assessed. AEs were reported by grouped terms or individual terms using the Medical Dictionary for Regulatory Activities, version 21.0 (Table S2). This report expands on previous reports of repotrectinib safety by characterizing time to AE onset and resolution, clinical expert opinion for AE management, medications used in AE management, underlying biological mechanisms of AEs, and patient subgroup analyses of AEs.

### Statistical analysis

The safety analysis was performed for the safety population and included all patients across all cohorts treated at the recommended dose. Data presented here were based on a cutoff date of October 15, 2023 and were summarized using descriptive statistics.

## Results

### Overall safety outcomes

**Figure 1:**
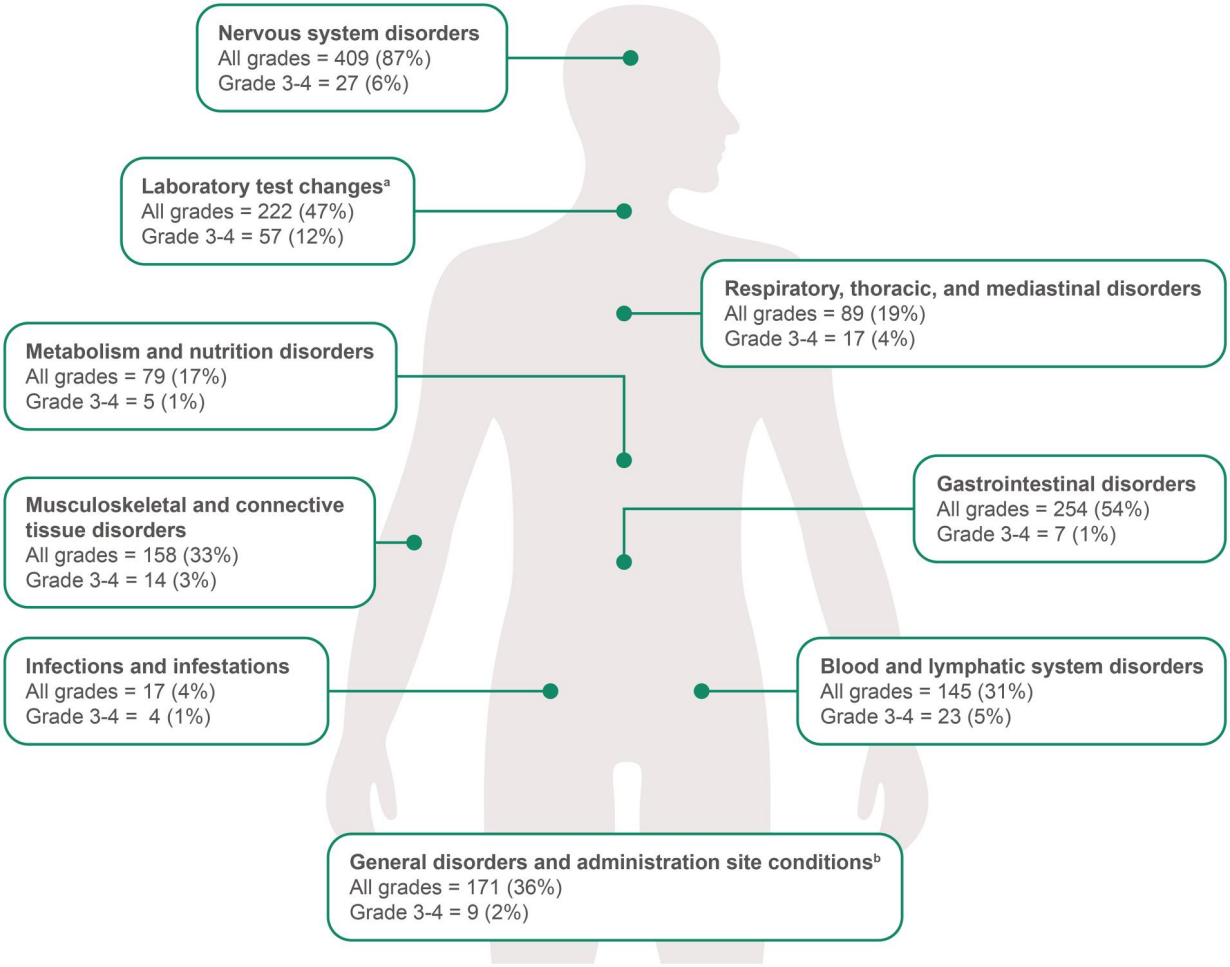
**Treatment-related adverse events by**  **system organ**  **class**. ^a^Laboratory test changes include increased alanine aminotransferase, increased aspartate aminotransferase, increased blood creatine phosphokinase, and increased weight. ^b^General disorders and administration site conditions include fatigue, pyrexia, and edema peripheral. Abbreviations: TEAE, treatment-emergent adverse event; TRAE, treatment-related adverse event.

The safety population included 472 patients, with a median follow-up of 23.9 months (range, 0.4–49.6). Baseline disease characteristics (Table S3), patient disposition and treatment exposure, including reasons for failure to increase dose to BID (Table 1), and incidences of the most common AEs by system organ class (Figure 1) are provided. Of 453 (96%) patients with any-grade treatment-related AEs (TRAEs), the most common were dizziness (58%), dysgeusia (50%), paraesthesia (31%), anemia (28%), and constipation (27%; Table 2). Grade ≥3 TRAEs were reported in 150 (32%) patients, serious TRAEs in 45 (10%), and fatal TRAEs in 2 (0.4%; included pulmonary embolism and sudden death). Treatment-emergent AEs (TEAEs) leading to dose interruption and dose reduction were reported in 261 (55%) and 199 (42%) patients, respectively (Table 3). Most frequent TEAEs leading to dose reduction included dizziness (*n* = 57; 12%) and ataxia (*n* = 32; 7%). In patients who reached the full study dose but had subsequent dose reduction due to AEs, dose reduction did not appear to negatively impact duration of response based on an exploratory landmark analysis (Figure S2). Re-escalation back to the full study dose was reported in 17 (10%) cases. Treatment discontinuation due to TEAEs was reported in 47 (10%) patients; the most common TEAEs included dyspnea (*n* = 5; 1%), pneumonitis (*n* = 5; 1%), and muscular weakness (*n* = 5; 1%).

**Table 1:** **Patient disposition and treatment exposure summary in all patients treated at the recommended dose**.

	All patients treated at the recommended dose (*N* = 472)
**Median relative dose intensity, % (range)^a^**	90 (12–101)
**Dose increased to 160 mg BID at day 15, n (%)^b^**	
** Yes**	404 (86)
** No**	66 (14)
** NA^c^**	2 (< 1)

a Relative dose intensity (%) is defined as (cumulative dose on study [in mg] divided by expected cumulative dose on study) times 100 where expected cumulative dose is defined as the starting dose times number of days on treatment. The expected cumulative dose is adjusted for patients who received a lead-in dose and for patients starting at BID dosing who are required to take the study drug QD on the first day.

b Percentages may not add up to 100% due to rounding.

c NA = patients who were not on treatment for at least 14 days.

Abbreviations: BID, twice daily; QD, once daily.

**Table 2:** Summary of TRAEs in all patients treated at the recommended dose.

TRAEs, *n* (%)	All grade	Grade 1	Grade 2	Grade 3	Grade 4	Grade 5
**Any TRAE**	453 (96)	100 (21)	203 (43)	137 (29)	11 (2)	2 (<1)
**TRAEs occurring in ≥10% of patients^a^**						
** Dizziness**	275 (58)	197 (42)	65 (14)	13 (3)	0	0
** Dysgeusia**	237 (50)	208 (44)	29 (6)	0	0	0
** Paraesthesia**	144 (31)	117 (25)	24 (5)	3 (1)	0	0
** Anemia**	133 (28)	43 (9)	71 (15)	19 (4)	0	0
** Constipation**	128 (27)	98 (21)	30 (6)	0	0	0
** Ataxia**	108 (23)	68 (14)	39 (8)	1 (<1)	0	0
** Increased aspartate aminotransferase**	92 (20)	78 (17)	7 (1)	6 (1)	1 (<1)	0
** Increased alanine aminotransferase**	90 (20)	70 (15)	13 (3)	7 (1)	0	0
** Increased blood creatine phosphokinase**	88 (19)	43 (9)	28 (6)	13 (3)	4 (1)	0
** Fatigue**	78 (17)	47 (10)	27 (6)	4 (1)	0	0
** Muscular weakness**	78 (17)	32 (7)	39 (8)	7 (1)	0	0
** Nausea**	57 (12)	43 (9)	12 (3)	2 (<1)	0	0
** Memory impairment**	56 (12)	51 (11)	4 (1)	1 (<1)	0	0
** Increased weight**	55 (12)	19 (4)	25 (5)	11 (2)	0	0
** Headache**	52 (11)	46 (10)	6 (1)	0	0	0
** Neuralgia**	51 (11)	40 (8)	9 (2)	2 (<1)	0	0
** Dyspnea**	48 (10)	21 (4)	24 (5)	2 (<1)	1 (<1)	0
**TRAEs that led to dose interruption**	182 (39)	3 (1)	78 (17)	93 (20)	8 (2)	0
**TRAEs that led to dose reduction**	179 (38)	26 (6)	98 (21)	51 (11)	4 (1)	0
**TRAEs that led to treatment discontinuation**	20 (4)	2 (<1)	10 (2)	6 (1)	2 (<1)	0
**Serious TRAEs**	45 (10)	0	11 (2)	28 (6)	4 (1)	2 (<1)
**Fatal TRAEs**	2 (<1)^b^	–	–	–	–	2 (<1)

a Based on individual terms.

b Fatal TRAEs included pulmonary embolism and sudden death.

Abbreviation: TRAE, treatment-related adverse event.

**Table 3:** Summary of TEAEs in all patients treated at the recommended dose.

TEAEs, *n* (%)	All grades	Grade 1	Grade 2	Grade 3	Grade 4	Grade 5
**Any TEAE**	469 (99)	39 (8)	161 (34)	204 (43)	37 (8)	28 (6)
**TEAEs occurring in ≥10% of patients^a^**						
** Dizziness**	299 (63)	212 (45)	74 (16)	13 (3)	0	0
** Dysgeusia**	250 (53)	219 (46)	31 (7)	0	0	0
** Constipation**	189 (40)	145 (31)	43 (9)	1 (< 1)	0	0
** Anemia**	186 (39)	55 (12)	93 (20)	37 (8)	1 (< 1)	0
** Paraesthesia**	165 (35)	134 (28)	28 (6)	3 (1)	0	0
** Dyspnea**	144 (31)	56 (12)	59 (13)	21 (4)	6 (1)	2 (< 1)
** Increased alanine aminotransferase**	117 (25)	89 (19)	18 (4)	9 (2)	1 (< 1)	0
** Fatigue**	112 (24)	65 (14)	42 (9)	5 (1)	0	0
** Ataxia**	111 (24)	68 (14)	41 (9)	2 (< 1)	0	0
** Increased aspartate aminotransferase**	110 (23)	86 (18)	10 (2)	13 (3)	1 (< 1)	0
** Muscular weakness**	106 (22)	49 (10)	47 (10)	10 (2)	0	0
** Headache**	97 (21)	85 (18)	11 (2)	1 (< 1)	0	0
** Increased blood creatine phosphokinase**	97 (21)	48 (10)	31 (7)	12 (3)	6 (1)	0
** Nausea**	96 (20)	72 (15)	19 (4)	5 (1)	0	0
** Cough**	88 (19)	70 (15)	17 (4)	1 (< 1)	0	0
** Diarrhea**	76 (16)	54 (11)	17 (4)	5 (1)	0	0
** Increased weight**	76 (16)	25 (5)	35 (7)	16 (3)	0	0
** Arthralgia**	74 (16)	57 (12)	16 (3)	1 (< 1)	0	0
** Memory impairment**	69 (15)	61 (13)	7 (1)	1 (< 1)	0	0
** Vomiting**	65 (14)	44 (9)	15 (3)	6 (1)	0	0
** Neuralgia**	63 (13)	50 (11)	11 (2)	2 (< 1)	0	0
** COVID-19**	62 (13)	35 (7)	20 (4)	6 (1)	1 (< 1)	0
** Peripheral edema**	60 (13)	48 (10)	12 (3)	0	0	0
** Decreased appetite**	58 (12)	44 (9)	12 (3)	2 (< 1)	0	0
** Disturbance in attention**	58 (12)	52 (11)	6 (1)	0	0	0
** Myalgia**	57 (12)	38 (8)	16 (3)	3 (1)	0	0
** Pain in extremity**	54 (11)	39 (8)	14 (3)	1 (< 1)	0	0
** Pyrexia**	53 (11)	34 (7)	15 (3)	4 (1)	0	0
** Somnolence**	52 (11)	44 (9)	8 (2)	0	0	0
** Back pain**	49 (10)	36 (8)	10 (2)	3 (1)	0	0
** Pneumonia**	48 (10)	9 (2)	13 (3)	24 (5)	0	2 (< 1)
** Decreased white blood cell count**	48 (10)	19 (4)	24 (5)	5 (1)	0	0
**TEAEs that led to dose interruption**	261 (55)	13 (3)	77 (16)	146 (31)	19 (4)	6 (1)
**TEAEs that led to dose reduction**	199 (42)	27 (6)	105 (22)	61 (13)	6 (1)	0
**TEAEs that led to treatment discontinuation**	47 (10)	2 (< 1)	10 (2)	20 (4)	5 (1)	10 (2)
**Serious TEAEs**	186 (39)	2 (< 1)	23 (5)	113 (24)	20 (4)	28 (6)
**Fatal TEAEs**	28 (6)	–	–	–	–	28 (6)

a Based on individual terms.

Abbreviation: TEAE, treatment-emergent adverse event.

The safety profile of repotrectinib was generally similar among ethnic (Asian and non-Asian patients; Table S4) and age subgroups (Table S5). There were numerically higher incidences of serious AEs, dose modifications, and treatment discontinuations observed in patients aged ≥75 years (*n* = 29); however, these findings should be interpreted with caution due to the small sample size. Incidences of AEs by gene fusion, treatment history, and prior TKI therapy were generally consistent (Tables S6–S8).

**Figure 2:**
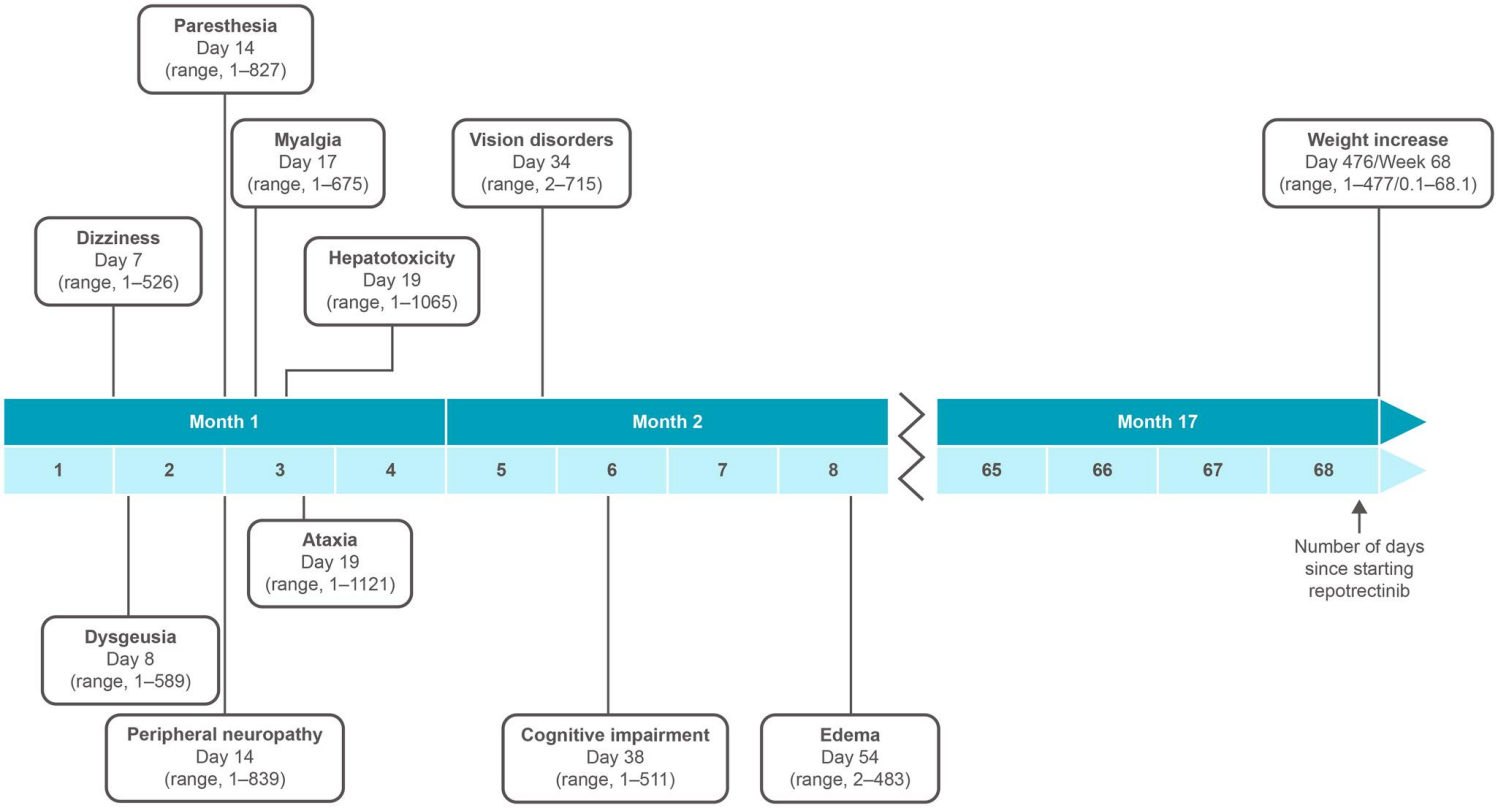
**Median time to first onset of select**  **TRAEs**. Abbreviation: TRAE, treatment-related adverse event.

Among all patients, 66 (14%) did not increase the dose to 160 mg BID (Table S9); reasons were based on protocol guidance: grade ≥3 TRAE (muscle cramps, urinary tract infection, and anemia), unmanageable grade ≥2 dizziness, ataxia, or paresthesia, or grade ≥3 clinically significant lab abnormalities (increased blood CPK, alanine aminotransferase, hepatic toxicity, low neutrophil count, and liver intolerance). CNS AEs were the most common reason for not increasing to BID dosing (*n* = 24 out of 66 patients [36%]). Median time to first onset of most TRAEs was ≤2 months after starting treatment (Figure 2).

### Dizziness and ataxia

Dizziness was the most common AE reported with repotrectinib. Dizziness is expected since TRK receptors are involved in nervous system development and maintenance.^10^^,^^17^ Patients described dizziness in a heterogeneous fashion as lightheadedness, imbalance, gait disturbance, and vertigo; these manifestations were episodic in select patients. Treatment-related dizziness (grouped term) was reported in 288 (61%) patients (Table S10), including 14 (3%) patients with grade ≥3 events. Dizziness as an individual term was reported in 275 (58%) of patients. Median time to first onset was 7 days (range, 1–526). Five (1%) patients with dizziness had neurogenic orthostatic hypotension. Median time to resolution of the first dizziness episode was 17.0 weeks (range, 0.1–191.7+) and 37.1 weeks (range, 0.1–191.7+) for the longest dizziness episode. Median time to resolution of the first and longest episodes of grade ≥3 dizziness were both 2.2 weeks (range, 0.3–94.4+).

Concomitant pharmacologic intervention without dose reduction or interruption was used per investigator discretion to manage treatment-related dizziness in 24 (8%) patients. The most common agents were meclizine (Bonine, Antivert), dimenhydrinate (Dramamine), and promethazine; QT-prolonging agents were prohibited per protocol. Of these 24 patients, 12 reported a downgrading of severity or resolution of dizziness. Dizziness due to neurogenic orthostatic hypotension can be treated with midodrine and fludrocortisone. Anecdotal prophylaxis treatment for dizziness was reported with meclizine (Bonine, Antivert), dimenhydrinate (Dramamine), and promethazine; however, their efficacy in preventing the emergence of dizziness could not be established based on previous reports.^18^ Patients should be advised to exercise caution when driving and operating large/hazardous machinery while taking repotrectinib.

Dose interruptions and reductions due to treatment-related dizziness (dizziness, dizziness postural, vertigo) occurred in 46 (10%) and 57 (12%) patients, respectively; no patient discontinued repotrectinib due to dizziness. Among 69 (24%) patients who received dose modification (reduction and/or interruption) for treatment-related dizziness, 54 (78%) reported downgrading or resolution of dizziness over time (Table S11). A total of 195 (68%) patients did not receive any dose modification or pharmacologic intervention to manage treatment-related dizziness, of whom 120 (62%) reported downgrading or resolution of dizziness.

Treatment-related ataxia (grouped term) or altered motor control was reported in 139 (29%) patients. Median times to first onset and resolution of any-grade treatment-related ataxia were 19 days (range, 1–1121) and 20.1 weeks (range, 0.6–151.4+), respectively. For grade ≥3 ataxia, median time to resolution was 2.9 weeks (range, 1.6–4.1). Ataxia developed concurrently with dizziness in 114 (24%) patients. Similar rates of CNS AEs, including dizziness and ataxia, were observed regardless of brain metastases at baseline (Table 4). Beyond dose modification, the most common management strategies for dizziness include vestibular suppressants and cautious positional changes (Table 5).

**Table 4:** **Incidence of CNS AEs by baseline brain metastases status^a^^,b^**.

CNS TEAEs, n (%)	Patients with brain metastases at baseline (*n* = 133)			Patients without brain metastases at baseline (*n* = 339)		
	Any grade	Grade 3	Grade 4	Any grade	Grade 3	Grade 4
	120 (90)	16 (12)	2 (2)	306 (90)	34 (10)	1 (< 1)
**Dizziness**	79 (60)	6 (8)	0	220 (65)	7 (4)	0
**Dysgeusia**	68 (52)	0	0	182 (54)	0	0
**Paraesthesia**	41 (31)	1 (1)	0	124 (37)	2 (1)	0
**Headache**	32 (24)	0	0	65 (19)	1 (1)	0
**Ataxia**	30 (23)	1 (1)	0	81 (24)	1 (1)	0
**Memory impairment**	22 (17)	0	0	47 (14)	1 (1)	0
**Neuralgia**	22 (17)	1 (1)	0	41 (12)	1 (1)	0
**Somnolence**	19 (15)	0	0	33 (10)	0	0
**Disturbance in attention**	18 (14)	0	0	40 (12)	0	0
**Cognitive disorder**	7 (5)	0	0	26 (8)	0	0
**Hypoesthesia**	6 (5)	0	0	12 (4)	1 (1)	0

a Based on TEAEs by individual terms.

b According to BICR.

Abbreviations: AE, adverse event; BICR, blinded independent central review; CNS, central nervous system; TEAE, treatment-emergent adverse event.

**Table 5:** Potential strategies beyond repotrectinib dose modification for AE management^a-d^.

Adverse events	Examples of alternative causes	Pharmacological interventions^e^	Nonpharmacologic management	Diagnostic procedures including specialized consult
**Ataxia and/or vertigo^17^^,^^29^^,^^30^**	• Brain metastases/leptomeningeal disease • Inner ear conditions	• Vestibular suppressants: antihistamines (eg, meclizine, dimenhydrinate), anticholinergics (eg, scopolamine) • Antiemetics to manage associated symptoms • Benzodiazepines (if severe symptoms only)	Physical therapy/vestibular rehabilitation	• Consultation with a neurologist can be considered • Safety evaluation/occupational driving evaluation
**Orthostatic dizziness or hypotension^17^^,^^31^^,f^**	• Orthostatic intolerance • Parkinson’s disease • Leptomeningeal disease/high intracranial pressure	Midodrine, fludrocortisone or droxidopa	• Cautious positional changes • Compression stockings, abdominal binders • Hydration	
**Withdrawal pain^17^^,^^32^**	• Disease progression • Arthritis	• Gradual, stepwise dose reduction and drug resumption if/when appropriate • Analgesics (nonsteroidal anti-inflammatory agents, opioids) • Gabapentinoids		Consultation with a pain team can be considered
**Weight gain^17^^,^^33^**	• Diet • Endocrine disorders • Metabolic disorders	• Glucagon-like peptide 1 analogs (eg, semaglutide, liraglutide, tirzepatide, exenatide) • Phentermine/topiramate • Lorcaserin • Naltrexone or bupropion • Metformin	• Lifestyle modification: dietary changes, exercise • Treatment of edema if present and contributory	Nutritionist, dietician, and/or weight loss specialist counseling
**Dysgeusia^34^**	• COVID-19 infection • Oral infection • Diabetes	• Zinc gluconate • Antidepressants	Dietary adjustments	Referral to a specialized smell and taste center can be considered
**Peripheral neuropathy^17^^,^^35^^,^^36^**	• Compression neuropathies • Radiculopathy • Diabetes • Vitamin deficiencies • Monoclonal gammopathy of undetermined significance • Hypothyroidism	• Anticonvulsants (eg, gabapentin, pregabalin) • Tricyclic antidepressants (eg, duloxetine) • Topical agents for pain relief (eg, lidocaine, capsaicin cream) • Non-opioid or opioid analgesics		Consultation with a neurologist can be considered

a A differential diagnosis should be performed to rule out any non-repotrectinib-related cause(s) of the adverse event and identify alternative etiologies.

b Consider potential occurrence of drug-to-drug interactions and medical comorbidities of patients, with careful weighing of potential benefits and risks, before initiating any pharmacological intervention in combination with repotrectinib.

c The potential strategies and their associated outcomes have not been prospectively assessed in TRIDENT-1.

d Refer to the AUGTYRO (repotrectinib) drug label and local guidelines for approved recommendations on management of AE; severe or long-lasting symptoms, potentially altering quality of life, and considered related to repotrectinib may require dose modification.^11^

e If appropriate, start pharmacological intervention at a lower dose and escalate if needed.

f Should this be observed.

### Withdrawal pain

Any-grade treatment-related withdrawal pain occurred in 6% of patients with treatment interruption for any reason (median time to onset, 2 days [range, 1–9]; median time to resolution, 2.3 weeks) and 5% of patients who discontinued repotrectinib permanently for any reason (median time to onset, 3 days [range, 2–9]; median times to resolution of any-grade and grade ≥3 events were 19.7 weeks [range, 0.6–106.1+] and 2.0 weeks [1.1–37.0+], respectively; Table S12). Any-grade treatment-emergent withdrawal pain occurred in 14% of patients with treatment interruption for any reason (median time to onset, 2 days [range, 1–39]; median times to resolution of any-grade and grade ≥3 withdrawal pain were 2.1 weeks [range, 0.1–146.0+] and 2.8 weeks [range, 0.4–5.1], respectively) and 12% of patients who discontinued repotrectinib permanently for any reason (median time to onset, 5 days [range, 2–24]). Median times to resolution of any-grade and grade ≥3 withdrawal pain were 19.7 weeks (0.1–106.1+) and 2.0 weeks (1.1–37.0+), respectively. Withdrawal pain has been previously reported in patients treated with TRK TKIs.^17^ TRK inhibition is associated with decreased nociception, and TRK TKI therapy withdrawal could result in heightened pain sensitivity. Patients have experienced quick resolution of withdrawal pain upon resuming TRK TKI. Based on expert experience, gradual reduction of TKI dose may also lower severity of or prevent withdrawal pain, such as reducing the dose by 40 mg every 3–5 days or as tolerated.^17^ Pharmacologic intervention (eg, gabapentin/pregabalin, steroids, and pain medication) may be considered during the withdrawal period as well as a prophylactic measure for patients with a history of repotrectinib withdrawal pain. Patients and caregivers should be informed that withdrawal pain may occur upon interrupting/stopping repotrectinib.

### Other neurologic AEs

Nervous system disorders (including dysgeusia, peripheral neuropathy, paresthesia, and cognitive impairment) were reported in 426 (90%) patients and are the most common AEs reported in patients treated with repotrectinib. Incidence of neurological AEs was similar regardless of brain metastases at baseline (Table 4). Nervous system disorders were generally mild (grade 1–2, 79%).

#### Dysgeusia

Treatment-related dysgeusia (grouped term) occurred in 257 (54%) patients (Table S13), with the median time to first onset of 8 days (range, 1–589). No grade ≥3 dysgeusia events were reported. Dysgeusia led to dose interruptions and reductions in 3 (1%) and 2 (<1%) patients, respectively. Median time to resolution of any-grade treatment-related dysgeusia was 75.4 weeks (range, 0.3–190.3+). No patient discontinued repotrectinib due to dysgeusia. Dysgeusia has no known pharmacological intervention resulting in improvement. Diet may be adjusted but may be of limited benefit.

#### Peripheral neuropathy

Treatment-related peripheral neuropathy (grouped term) occurred in 82 (17%) patients (Table S14), with the median time to first onset of 14 days (range, 1–839). Peripheral neuropathy was mostly low grade; grade ≥3 treatment-related events occurred in 5 (1%) patients. Peripheral neuropathy led to dose interruptions and reductions in 7 (1%) and 6 (1%) patients, respectively. Among 8 patients with any dose modification, 6 (75%) patients reported downgrading or resolution of peripheral neuropathy. Median times to resolution of any-grade and grade ≥3 treatment-related peripheral neuropathy were 12.1 weeks (range, 0.1–149.7+) and 2.0 weeks (range, 0.4–37.0+), respectively. Discontinuation of repotrectinib due to peripheral neuropathy was reported in 1 patient. Gabapentin, duloxetine, and similar agents may be used to manage peripheral neuropathy.

#### Paresthesia

Treatment-related paresthesia (grouped term) occurred in 166 (35%) patients (Table S15), with median time to first onset of 14 days (range, 1–827). Most paresthesia were low grade in severity; treatment-related grade ≥3 events occurred in 4 (1%) patients. Paresthesia led to dose interruptions and reductions in 8 (2%) and 13 (3%) patients, respectively. Median times to resolution of any-grade and grade ≥3 events of treatment-related paresthesia were 16.4 weeks (range, 0.1–190.3+) and 11.5 weeks (range, 2.0–20.6+), respectively. No patient discontinued repotrectinib due to paresthesia. Patients experiencing paresthesia should be monitored for severity and impact on lifestyle. Standard supportive care measures for paresthesia and/or dose reduction may be considered for significant symptoms.

#### Cognitive impairment

Treatment-related cognitive impairment (grouped term) occurred in 89 (19%) patients (Table S16), with the median time to onset of 38 days (range, 1–511). Cognitive impairment was mostly low grade; treatment-related grade ≥3 cognitive impairment occurred in 3 (1%) patients. Cognitive impairment led to dose interruption and reduction in 9 (2%) and 11 (2%) patients, respectively. Among 12 patients with any dose modification, 9 (75%) experienced downgrading or resolution of treatment-related cognitive impairment over time. Median times to resolution for any-grade and grade ≥3 events were 58.6 (range, 0.3–170.4+) and 2.6 weeks (range, 1.0–3.0), respectively. Discontinuation of repotrectinib due to cognitive impairment was reported in 3 (1%) patients. Patients and caregivers should be educated about this potential side effect. Patients should be monitored closely, and follow-ups should include caregivers, as patients may have impaired insight. Dose modification is suggested for moderate or severe cognitive impairment. A neurology/psychiatry referral may be helpful in certain cases.

### Myalgia/increased CPK

Treatment-related myalgia (grouped term) occurred in 77 (16%) patients. Median time to first onset of treatment-related myalgia was 17 days (range, 1–675). CPK levels were assessed when patients presented with symptoms of myalgia. Increased CPK level occurred in 88 (19%) patients; no cases of rhabdomyolysis were reported. Myalgia and increased level of CPK were mild; 12 (2.5%) and 18 (4%) patients reported grade ≥3 events, respectively. Myalgia led to dose interruption in 7 (9%) patients; no patient had dose reduction. Of 11 patients with any dose modification, 9 (82%) reported downgrading or resolution of myalgia. Patients should be monitored for this side effect. Pain medication and, in cases with substantial CPK elevation, hydration may be considered. Among the 48 (62%) patients who had resolution of treatment-related myalgia, median times to resolution of any-grade and grade ≥3 events were 12.1 (range, 0.7–187.1+) and 1.6 weeks (range, 0.6–88.0+), respectively. Of note, some incidences of cancer-associated pain may be misreported as myalgia; muscle exercise may be a confounding factor in asymptomatic CPK elevation.

### Pneumonitis/ILD

Symptoms of pneumonitis (grouped term) are usually described as increasing breathlessness and cough. Treatment-related pneumonitis occurred in 13 (3%) patients (Table S17). Pneumonitis was mild in severity; 4 (1%) patients reported grade ≥3 treatment-related pneumonitis. No patient with pneumonitis/ILD received prior thoracic radiotherapy; 2 patients received prior immunotherapy. Pneumonitis led to dose interruption and dose reduction in 8 (2%) and 3 (1%) patients, respectively. Among 7 patients with any dose modification, 6 (86%) reported downgrading or resolution of treatment-related pneumonitis. Five (1%) patients discontinued repotrectinib treatment due to pneumonitis. Corticosteroids and potentially steroid-sparing anti-inflammatory agents should also be considered for managing ILD.^19^

### Weight increase

Preclinical studies and congenital syndromes showed that reducing TRK signaling through TRK inhibition can result in hyperphagia^17^ and weight gain. Treatment-related weight increase occurred in 55 (12%) patients. Weight increase was mostly low grade (5% to <20% from baseline per the CTCAE), and 11 (2%) patients reported grade 3 treatment-related weight increase (≥20% from baseline). Median time to first onset of treatment-related weight increase was 68 weeks (range, 0.1–68.1). No patient reported dose reduction, treatment interruption, or discontinuation of repotrectinib due to weight gain. Median times to resolution of any-grade and grade ≥3 events were 104.1 weeks (range, 2.1–175.1+) and 72.1 weeks (range, 20.1–74.1+), respectively. Patients should be advised that weight gain may occur; weight should be recorded at every clinic visit and logged by patients at home. Weight increase that does not adversely impact quality of life (QOL) may not warrant intervention provided substantial increase above ideal body weight is not seen; however, lifestyle recommendations (ie, diet and exercise), referral to specialists (ie, endocrinologist), and dose modification may be helpful.^17^^,^^20^ Per physician discretion, monotherapy or combination therapy with GLP-1 analogs, metformin, bupropion, topiramate, sibutramine, and phentermine may be used.^21^ Drug reimbursement may be challenging, and drug transit times through the gut may be affected by GLP-1 analogs; the impact of the latter on repotrectinib absorption warrants further investigation. Concurrent weight increase and edema may occur; edema should be managed first as it may influence weight.

### Fractures

Treatment-emergent fractures (grouped term) occurred in 15 (3%) patients, one (<1%) of which was considered treatment-related per investigator assessment (Table S18). The most commonly reported AE was foot fracture in 3 (1%) patients. Incidence of fractures was similar among adult patients of all ages. Fractures led to dose interruption in 3 (1%) patients. Discontinuation of repotrectinib was reported in 1 (<1%) patient with a femur fracture. Of patients with fractures, 8 (53%) reported concurrent CNS TEAEs (7 patients reported dizziness, 2 reported paraesthesia). Treating medical team, patients, and caregivers should monitor for symptoms or signs of potential fractures, such as pain, deformity, or change in mobility. Standard management and orthopedic consultation should be considered when necessary. The mechanism for fractures following repotrectinib treatment is not well understood; pediatric patients may have higher risk, as shown in separate trials with TRK TKIs.^22^^,^^23^

### Other relevant select AEs

Treatment-related edema (grouped term) occurred in 43 (9%) patients, with median time to first onset of 54 days (range, 2–483). No grade ≥3 edema events were reported. Of 19 patients who reported resolution of edema, median time to resolution was 100.0 weeks (range, 1.1–162.9+). No patient reported dose reduction, treatment interruption, or discontinuation of repotrectinib due to edema. Patients should be assessed for comorbidities that may cause edema, such as underlying heart disease or renal/thyroid dysfunction.^20^ Edema can be managed with nonpharmacologic strategies (see Supplementary Results) or with diuretics such as furosemide, as recommended by experts. For persistent edema or edema impacting QOL, other causes should be considered or referral to specialists may be required.^20^ Repotrectinib dose may be interrupted until symptoms improve then reinitiated at a reduced dose.^11^

Treatment-related hepatotoxicity (grouped term) was reported in 114 (24%) patients, with median time to first onset of 18.5 days (range, 1–1065). Hepatotoxicity was mostly low grade; 12 (3%) patients reported grade ≥3 treatment-related events. Hepatotoxicity led to dose interruption and reduction in 17 (4%) and 7 (1%) patients, respectively. Among 11 patients with any dose modification, 9 (82%) reported downgrading or resolution of treatment-related hepatotoxicity. Median times to resolution of any-grade events were 4.3 weeks (range, 1.0–63.7+), and 3.3 weeks (range, 1.1–20.1) for grade ≥3 events. No patient discontinued repotrectinib treatment due to hepatotoxicity.

Treatment-related vision disorders (grouped term) were reported in 31 (7%) patients (Table S19), with the median time to first onset of 34 days (range, 2–715). Vision disorders were mostly low grade, and none were sight-threatening; grade ≥3 treatment-related events occurred in 1 (<1%) patient. Vision disorders led to dose interruption and dose reduction in 7 (1%) patients and 1 (0.2%) patient, respectively. Discontinuation of repotrectinib due to vision disorders was reported in 1 (0.2%) patient with color blindness.

Other relevant AEs are described in Supplementary Results, including edema, QTc prolongation, hyperbilirubinemia, increased blood bilirubin level, hyperuricemia, and increased blood uric acid. Additional potential strategies for AE management are provided in Table 5.

## Discussion

This manuscript features the most comprehensive characterization of side effects observed with repotrectinib, a ROS1 and TRK inhibitor approved in multiple regulatory environments. Grade ≥3 events for certain AEs such as ataxia appeared to resolve more quickly than lower-grade events, which may reflect more aggressive management mandated in the protocol. Certain medications should not be used concomitantly with repotrectinib (Supplementary Discussion).

Dizziness, the most common TRAE (58%), often occurred ≤7 days of therapy while patients were on QD dosing, although late-onset dizziness was observed. No factors, including baseline brain metastases, appeared to strongly determine which patients developed dizziness, underscoring that patient sensitivity to TRK inhibition may not be easily predicted. The type of dizziness (eg, vertigo, imbalance, etc) must be characterized carefully as pharmacologic intervention (for which efficacy remains uncertain) should be tailored to pathophysiology (ie, meclizine for vertigo versus midodrine for postural hypotension). Since we observed that more definitive terms (ie, postural dizziness, vertigo, nystagmus) were not commonly used by investigators, and terms with potential overlap (ie, dizziness and ataxia) could not be easily disentangled, we suggest that future TRK inhibitor trials require concrete descriptions of individual terms in this category beyond CTCAE descriptions, including questionnaires probing the type of dizziness (vertigo/gait disturbance versus lightheadedness/orthostasis) and ataxia (cerebellar versus sensory), and/or a neurology consult. The pervasiveness of dizziness and its effect on QOL (including grade 1 events) should be discussed with patients even though no patients discontinued repotrectinib for dizziness and prior reports established that most patients treated with repotrectinib experienced stable or improved health-related QOL, as assessed by functional, symptom, and global health status/QOL scales (Supplementary Discussion).^24^^,^^25^

Because repotrectinib may affect the nervous system, it is important to note that neuropathy (occurring in 17% of patients and which may occur with chemotherapy) was much less frequently observed than dizziness. Treatment-related paresthesia, including dysesthesia, a burning skin sensation, and formication, was more common (occurring in 35% of patients) than neuropathy. The majority of both events were low grade, and rates of dose modification were low (1–3%), suggesting less QOL impact; however, side effects could have overlapped, with dizziness potentially overshadowing other AEs. Patients and caregivers should also monitor cognitive impairment, although severe treatment-related events were rare (1%). Additionally, withdrawal pain was observed in some patients when temporarily or permanently discontinuing repotrectinib (similar to other TRK inhibitors) and should be considered by providers when performing procedures on or caring for patients who may need TKI interruption. Weight gain due to hyperphagia should be monitored as this effect can insidiously increase weight over time.

Compared with other TRK inhibitors entrectinib and larotrectinib, repotrectinib appears to be associated with a higher incidence of any-grade treatment-related dizziness, dysgeusia, paresthesia, anemia, ataxia, increased blood CPK, muscular weakness, memory impairment, headache, neuralgia, and dyspnea.^26^^,^^27^ However, any-grade treatment-related constipation, increased aspartate aminotransferase, increased alanine aminotransferase, fatigue, nausea, increased weight, diarrhea, vomiting, arthralgia, dysphagia, and decreased white blood cell count appear to occur at higher rates with entrectinib and/or larotrectinib. Any-grade treatment-related myalgia and treatment-emergent fractures are reported at similar rates. Rates of grade 3-4 TRAEs were low across repotrectinib, entrectinib, and larotrectinib. Compared with taletrectinib, a multikinase TKI with ROS1 selectivity, taletrectinib appears to have higher rates of any-grade treatment-related increased aspartate aminotransferase, increased alanine aminotransferase, nausea, vomiting, and QT prolongation.^28^ Rates of dizziness and dysgeusia appeared higher with repotrectinib, and rates of anemia, constipation, and increased blood CPK appeared similar between repotrectinib and talectrectinib.^28^ Notably, cross-trial comparisons should be interpreted with caution as the study protocols, patient populations, and safety data reporting differ.

The ongoing TRIDENT-3 trial (NCT06140836) is assessing outcomes of repotrectinib versus crizotinib, a first-generation ALK and ROS1 TKI, in TKI-naïve patients with *ROS1*+ NSCLC. Further, a study investigating repotrectinib in pediatric and young adult patients (CARE; NCT04094610) with *ROS1*+ or *NTRK*+ locally advanced/metastatic solid tumors is ongoing; preliminary data suggest a consistent safety profile between adult and pediatric patients.^11^^,^^22^ Studies of ROS1/TRK TKIs should prospectively capture AEs in granular detail to provide better guidance for managing these AEs reported in the clinics.

## Supplementary Material

oyag137_Supplementary_Data

## Data Availability

Bristol Myers Squibb company policy on data sharing may be found at https://www.bms.com/researchers-and-partners/independent-research/data-sharing-request-process.html.
