# Supplementary material for: Characterization and clinical management of adverse events following treatment with repotrectinib: a TRIDENT-1 analysis
Source: Oncologist. 2026 Apr 16;31(6):oyag137. doi: 10.1093/oncolo/oyag137 (PMC13153690; doi:10.1093/oncolo/oyag137)
Supplement: oyag137_Supplementary_Data [file oyag137_supplementary_data.pdf]

SUPPLEMENTARY APPENDIX

SUPPLEMENTARY METHODS 3

SUPPLEMENTARY RESULTS 4

SUPPLEMENTARY DISCUSSION 6

SUPPLEMENTARY FIGURES AND TABLES 8

Figure S1. Overview of the Phase 1/2 TRIDENT-1 study design 8

Figure S2. Landmark analysis of duration of response per BICR by dose reduction in patients with *ROS1*+ NSCLC 9

Table S1. AE-specific dose modification recommendations according to the TRIDENT-1 study protocol[a] 11

Table S2. Summary of grouped and individual terms[a] 14

Table S3. Patient and disease characteristics at baseline 16

Table S4. Summary of AEs in all patients treated at the recommended dose in Asian and non-Asian patients 18

Table S5. Summary of AEs in all patients treated at the recommended dose by age 19

Table S6. Summary of AEs in all patients treated at the recommended dose in the *ROS1*+ NSCLC and *NTRK*+ solid tumor populations 20

Table S7. Summary of AEs in all patients treated at the recommended dose by prior treatment history 21

Table S8. Summary of AEs in all patients treated at the recommended dose by prior TKI therapy 23

Table S9. Summary of reasons for patients in the safety population who did not dose increase to 160 mg BID 25

Table S10. Incidence of dizziness and ataxia by individual terms in all patients treated at the recommended dose 27

Table S11. Subsequent downgrading with resolution of TRAEs after dose modification in all patients treated at the recommended dose[a] 30

Table S12. Summary of withdrawal pain in all patients treated at the recommended dose 32

Table S13. Incidence of dysgeusia by individual terms in all patients treated at the recommended dose 34

Table S14. Incidence of peripheral neuropathy by individual terms in all patients treated at the recommended dose 36

Table S15. Incidence of paresthesia by individual terms in all patients treated at the recommended dose 38

Table S16. Incidence of cognitive impairment by individual terms in all patients treated at the recommended dose 40

Table S17. Incidence of pneumonitis by individual terms in all patients treated at the recommended dose 43

Table S18. Incidence of fractures by individual terms in all patients treated at the recommended dose 44

Table S19. Incidence of vision disorders by individual terms in all patients treated at the recommended dose 46

REFERENCES 49

SUPPLEMENTARY METHODS

Duration of response per blinded independent central review (BICR) was assessed by dose reduction in *ROS1* fusion–positive (*ROS1*+) non-small cell lung cancer (NSCLC) using an exploratory 3-month landmark Kaplan–Meier analysis; 95% CIs were calculated using Greenwood’s formula for variance estimation and constructed on the log-log scale.

SUPPLEMENTARY RESULTS

Additional information on other relevant select adverse events (AEs) is summarized.

Edema

Nonpharmacological management strategies: Low grade edema may be resolved with therapeutic interventions such as physiotherapy, lymphatic drainage massage, and compression stockings. Leg elevation and lifestyle modification such as increased exercise and limiting dietary salt intake may also be effective and should also be considered prior to dose modification.

QTc prolongation

QTc prolongation occurred in 5 (1%) patients and was considered treatment related in 2 (<1%) patients. No grade ≥3 events were reported. No events of QTc prolongation led to dose modification or treatment discontinuation. Based on pharmacokinetic/pharmacodynamic, clinical, and nonclinical data and external expert consultation, there is no evidence of clinically relevant effects of repotrectinib on cardiac repolarization (F), heart rate, PR interval, or QRS duration. This observation potentially broadens the drugs that could be used for supportive care. Normal QTcF in men is 450 ms and in women is 470 ms. If the QTcF is greater than the respected normal readings, it is considered an AE, and if it is > 60 ms from baseline, it is considered clinically meaningful.[1] Patients should be informed of potential risk for atrioventricular block and contact their healthcare providers if they experience chest pain, changes in heartbeat, palpitations, dizziness, lightheadedness, or fainting, as well as changes in new heart or blood pressure medication. Monitoring QTc prolongation with electrocardiogram at baseline, month 1, and as clinically relevant thereafter is recommended.

Hyperbilirubinemia and increased blood bilirubin level

Events of treatment-emergent hyperbilirubinemia and increased blood bilirubin level were reported in 1 (< 1%) and 8 (2%) patients, respectively. No patient reported treatment-related hyperbilirubinemia and increased blood bilirubin levels was reported in 1 (< 1%) patient. Grade ≥ 3 treatment-emergent and treatment-related increased blood bilirubin were reported in 3 (1%) patients and 1 (< 1%) patient, respectively. Increased blood bilirubin led to dose interruption and dose reduction in 2 patients and 1 patient, respectively, and no patient discontinued repotrectinib treatment.

Hyperuricemia and increased blood uric acid

Treatment-emergent hyperuricemia and increased blood uric acid occurred in 22 (5%) and 5 (1%) patients, respectively. Treatment-related hyperuricemia and increased blood uric acid were reported in 12 (3%) and 3 (1%) patients, respectively. Dose reduction due to hyperuricemia was reported in 1 patient, and no patient reported dose interruption or treatment discontinuation. Hyperuricemia prophylaxis is generally not indicated.

SUPPLEMENTARY DISCUSSION

Patient-reported outcomes

Patient-reported outcomes (PROs) from TRIDENT-1 were previously reported for patients with *ROS1*+ advanced or metastatic NSCLC and patients with *NTRK* fusion–positive (*NTRK*+) locally advanced or metastatic solid tumors.[2,3] To briefly summarize, quality of life (global health status [GHS]/QOL) were assessed by the European Organisation for Research and Treatment of Cancer (EORTC) Quality of Life core questionnaire (QLQ-C30) at screening, pre-dose day 1 of each treatment cycle (month), and at the end-of-treatment visit.[2,3] Treatment-related symptoms for patients with NSCLC were also assessed by the lung cancer module (QLQ-LC13).[2]

Across the majority of the 156 patients with *ROS1*+ NSCLC included in the PRO analysis (63 TKI-naïve and 93 TKI-pretreated patients), mean changes over time remained stable or improved in most scores.[2] The median time to first improvement in GHS/QOL was 3.71 months for TKI-naïve and 4.67 months for TKI-pretreated patients.[2] For key lung cancer symptoms (cough, pain in chest, and hemoptysis), improvements were observed; the median time to first improvement for cough was 1.84 months for TKI-naïve and 2.89 months for TKI-pretreated patients.[2]

A total of 79 patients with *NTRK*+ solid tumors were included in the PRO analysis (35 TKI-naïve and 44 TKI-pretreated patients).[3] Among patients assessed at cycle 12, 52.9% of TKI-naïve patients and 78.6% of TKI-pretreated patients had stable or improved responses in GHS/QOL.[3] The median time to definitive deterioration for the GHS/QOL was 17.5 and 13.1 months for the TKI-naïve and TKI-pretreated cohorts, respectively.[3] The median time to first improvement in GHS/QOL was not reached in either cohort.[3]

Concomitant medications

Certain medications should not be used concomitantly with repotrectinib

1. Use of strong CYP3A inhibitors is prohibited as these inhibitors may increase repotrectinib exposure leading to a potential increase in toxicities (ie, boceprevir, clarithromycin, cobicistat, danoprevir and ritonavir, elvitegravir and ritonavir, grapefruit juice or grapefruit/grapefruit-related citrus fruits [eg, Seville oranges, pomelos], idelalisib, indinavir and ritonavir, itraconazole, ketoconazole, lopinavir and ritonavir, nefazodone, nelfinavir, paritaprevir and ritonavir and (ombitasvir and/or dasabuvir), posaconazole, ritonavir, saquinavir and ritonavir, telaprevir, telithromycin, tipranavir and ritonavir, troleandomycin, voriconazole)
2. Use of sensitive CYP3A4 or CYP2B6 substrates should be avoided as repotrectinib may induce CYP3A4 or CYP2B6 activity in the liver, which may decrease exposures of coadministered drugs leading to the potential decrease in efficacy of these drugs
    a. Sensitive CYP3A substrates: alfentanil, avanafil, budesonide, buspirone, conivaptan, darifenacin, darunavir, dasatinib, dronedarone, ebastine, eletriptan, eplerenone, everolimus, felodipine, ibrutinib, indinavir, lomitapide, lovastatin, lurasidone, maraviroc, midazolam, naloxegol, nisoldipine, quetiapine, saquinavir, simvastatin, sildenafil, sirolimus, tacrolimus, ticagrelor, tipranavir, tolvaptan, triazolam, vardenafil
    b. Sensitive CYP2B6 substrates: bupropion, efavirenz

SUPPLEMENTARY FIGURES AND TABLES

Figure S1. Overview of the Phase 1/2 TRIDENT-1 study design

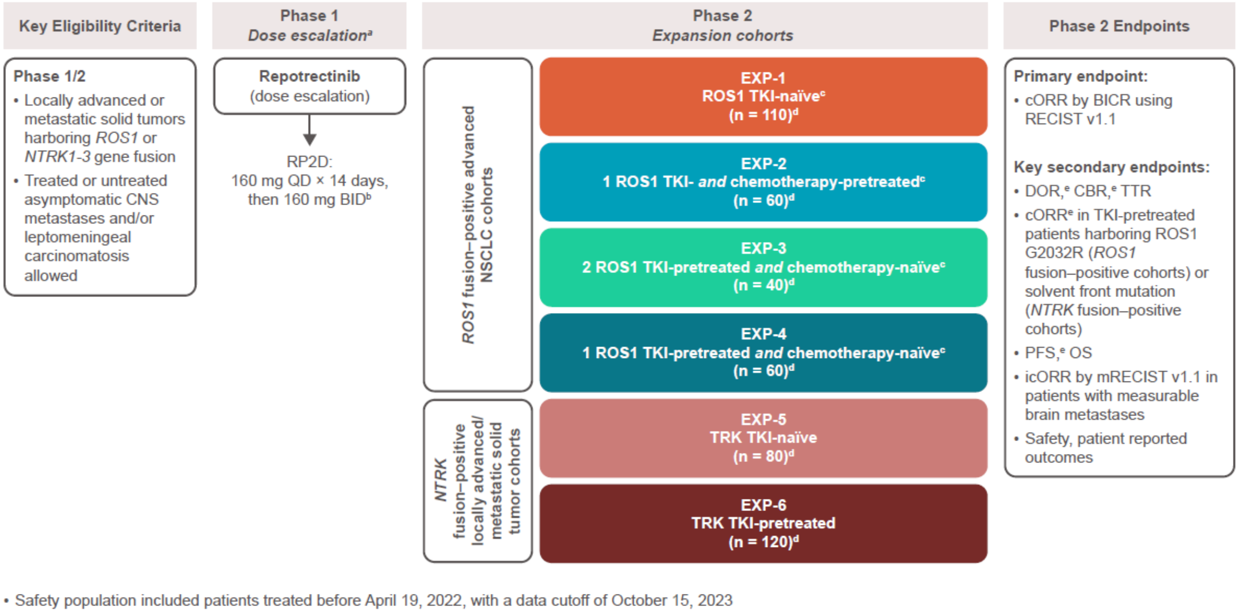

[a]Phase 1 primary endpoints: DLT, MTD, RP2D.

[b]Based on tolerability.

[c]Up to 1 prior line of chemotherapy or immunotherapy is allowed for EXP-1; must have received 1 prior line of platinum-based chemotherapy immunotherapy before or after a ROS1 inhibitor for EXP-2; no prior lines of chemotherapy or immunotherapy are allowed for EXP-3 and EXP-4.

[d]N's for expansion cohort indicate enrollment targets.

[e]By RECIST v1.1.

Abbreviations: BICR, blinded independent central review; BID, twice daily; CBR, clinical benefit rate; CNS, central nervous system; cORR, confirmed objective response rate; DLT, dose-limiting toxicity; DOR, duration of response; icORR, intracranial objective response rate; mRECIST, modified Response Evaluation Criteria in Solid Tumors; MTD, maximum tolerated dose; NSCLC, non-small cell lung cancer; OS, overall survival; PFS, progression-free survival; QD, once daily; RECIST, Response Evaluation Criteria in Solid Tumors; RP2D, recommended phase 2 dose; TKI, tyrosine kinase inhibitor; TTR, time to response.

Figure S2. Landmark analysis of duration of response per BICR by dose reduction in patients with *ROS1*+ NSCLC

Duration of response is shown in patients with *ROS1*+ advanced NSCLC who escalated to 160 mg BID stratified by subsequent dose reduction due to adverse event within 3 months.[a,b] ROS1 TKI-naïve patients (A) and patients with 1 prior ROS1 TKI and no prior chemo (B). DOR in the *NTRK*+ cohorts was previously presented.[4]

A

ROS1 TKI-naïve patients	No. events/ censored	Median duration of response (95% CI) *mo*
— With dose reduction due to AE within 3 mo	6 / 17	33.6 (12.9–NE)
---- Without dose reduction due to AE or with dose reduction after 3 mo	8 / 26	NR (20.3–NE)

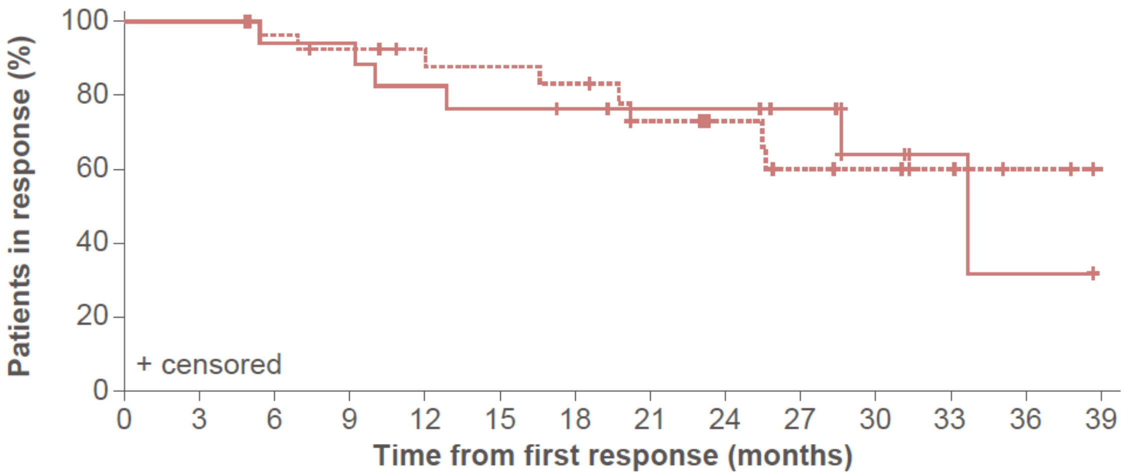

No. at risk

— With dose reduction due to AE within 3 mo	17	17	16	16	14	13	12	10	10	8	4	2	1	0
---- Without dose reduction due to AE or with dose reduction after 3 mo	26	26	24	22	20	19	18	14	11	8	7	5	2	0

B

Patients with 1 prior ROS1 TKI and no prior chemo	No. events/ censored	Median duration of response (95% CI) *mo*
— With dose reduction due to AE within 3 mo	2 / 4	NR (5.6–NE)
---- Without dose reduction due to AE or with dose reduction after 3 mo	10 / 14	13.5 (5.2–NE)

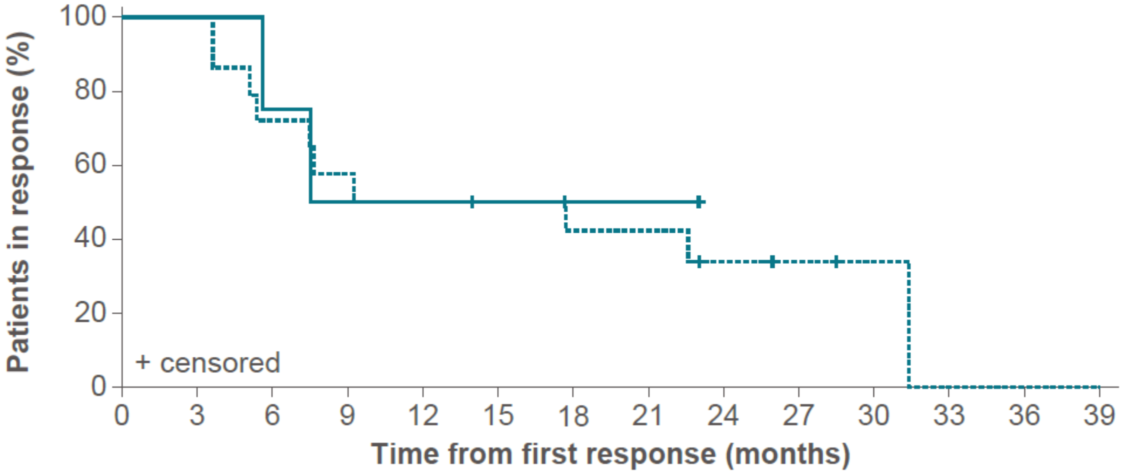

No. at risk

**— With dose reduction due to AE within 3 mo**													
4	4	3	2	2	2	1	1	0	0	0	0	0	0
**---- Without dose reduction due to AE or with dose reduction after 3 mo**													
14	14	10	8	7	6	5	5	3	2	1	0	0	0

[a]The calculations were performed using a 3-month landmark analysis to mitigate immortal time bias. Results should be interpreted with caution due to the exploratory nature of this analysis, which may be impacted by confounding factors.

[b]Of the 21 patients who reached the 160 mg BID dose and dose-reduced due to an AE in the first 3 months, 11 dose-reduced to 120 mg. Of those, 6 had a second dose reduction (4 patients to 80 mg BID and 2 patients to other doses). Other patients followed different patterns of dose reduction, most commonly reducing from 160 mg BID to 160 mg QD or 120 mg QD.

Abbreviations: AE, adverse event; BICR, blinded independent central review; BID, twice daily; DOR, duration of response; NE, not evaluable; NR, not reached; NSCLC, non-small cell lung cancer; QD, once daily; *ROS1*+, *ROS1* fusion–positive; TKI, tyrosine kinase inhibitor.

Table S1. AE-specific dose modification recommendations according to the TRIDENT-1 study protocol[a]

Toxicity	CTCAE grade 1	CTCAE grade 2	CTCAE grade 3	CTCAE grade 4
Hematologic	Continue at same dose level	Continue at same dose level	Withhold dose until toxicity is grade ≤ 2, or has returned to baseline, then resume treatment at the same dose level or reduce by 1 dose level as per the investigator's discretion<br><br>Grade 3 lymphopenia without other dose-limiting events (eg, opportunistic infection) may continue study treatment without interruption	Withhold dose until toxicity is grade ≤ 2, or has returned to baseline, reduce by 1 dose level and resume treatment<br><br>Grade 4 lymphopenia without other dose-limiting events (eg, opportunistic infection) may continue study treatment without interruption
Nonhematologic AEs excluding dizziness, ataxia, and paresthesia	Continue at same dose level	Continue at same dose level<br><br>For prolonged or intolerable CNS toxicity, withhold dose until toxicity is grade ≤ 1 or has returned to baseline, then reduce by 1 dose level and resume treatment	Withhold dose until toxicity is grade ≤ 1 or has returned to baseline, then reduce by 1 dose level and resume treatment	Withhold dose until toxicity is grade ≤ 1 or has returned to baseline, then reduce by 1 dose level and resume treatment; or discontinue treatment as per the investigator's discretion

Dizziness, ataxia, and paresthesia	Continue at same dose level	Withhold current dose until toxicity is grade ≤ 1 or has returned to baseline OR reduce dose by 1 dose level immediately	Withhold dose until toxicity is grade ≤ 1 or has returned to baseline, then reduce by 1 dose level and resume treatment	Withhold dose until toxicity is grade ≤ 1 or has returned to baseline, then reduce by 1 dose level and resume treatment; or discontinue treatment as per the investigator's discretion after discussion with Medical Monitor
Pneumonitis (in the absence of disease progression, pulmonary embolism, positive cultures, or radiation effect)	Asymptomatic, radiographic findings only: no need for dose adjustment. Initiate appropriate monitoring<br><br>Symptomatic: withhold current dose until toxicity has returned to baseline. Rule out infection and consider initiating treatment with corticosteroids. Then resume treatment at the same dose. Discontinue permanently if pneumonitis recurs or if failure to recover after 6 weeks of study treatment hold and steroid treatment	Withhold current dose until toxicity has returned to baseline. Rule out infection and consider initiating treatment with corticosteroids. Then resume treatment at 1 dose level lower<br><br>Discontinue permanently if pneumonitis recurs or if failure to recover after 6 weeks of study treatment hold and steroid treatment	Discontinue treatment	Discontinue treatment

Prolonged QTc	Assess electrolytes and concomitant medications<br><br>Correct any electrolyte abnormalities or hypoxia<br><br>Continue at the same dose level	Assess electrolytes and concomitant medications<br><br>Correct any electrolyte abnormalities or hypoxia<br><br>Continue at the same dose level	Withhold dose<br><br>Assess electrolytes and concomitant medications<br><br>Correct any electrolyte abnormalities or hypoxia<br><br>Upon recovery to grade ≤ 1: if no other cause for QTc prolongation is found or is considered drug related, resume treatment at 1 dose level lower	Discontinue treatment

Note: Physicians should consult with local prescribing information for recommendations on dose modifications to manage AEs.

[a]Graded per CTCAE v4.03.

Abbreviations: AE, adverse event; CNS, central nervous system; CTCAE, Common Terminology Criteria for Adverse Events.

Table S2. Summary of grouped and individual terms[a]

Grouped term	Individual terms
Dizziness	Dizziness, vertigo, dizziness postural, dizziness exertional, ataxia, and vertigo positional
Ataxia	Ataxia, gait disturbance, balance disorder, cerebellar ataxia, coordination abnormal, and nystagmus
Dysgeusia	Dysgeusia, taste disorder, ageusia, sensory disturbance, allodynia, hypogeusia, and sensory loss
Peripheral neuropathy	Neuralgia, peripheral sensory neuropathy, neuropathy peripheral, peripheral motor neuropathy, and polyneuropathy
Paresthesia	Paresthesia, hypoesthesia, hyperesthesia, dysesthesia, burning sensation, anesthesia, and formication
Myalgia	Myalgia, pain in extremities, arm pain, hand pain, leg pain, back pain, neck pain, muscle spasm, calf pain, and myopathy
Cognitive impairment	Memory impairment, disturbance in attention, attention deficit hyperactivity disorder, cognitive disorder, confusional state, delirium, amnesia, aphasia, altered state of consciousness, depressed level of consciousness, neurological decompensation, bradyphrenia, delusion, dysgraphia, hallucination, and mental status changes
Pneumonitis	Pneumonitis, interstitial lung disease
Fractures	Fracture; foot, rib, spinal compression, acetabulum, ankle, femur, fibula, forearm, sternal, and upper limb fractures
Edema	Edema, swelling, puffiness, enlargement, and inflammation

Hepatoxicity	Increased alanine aminotransferase, increased aspartate aminotransferase, hypertransaminasemia, hepatic cytolysis, and increased transaminases
Vision disorders	Blurred vision, visual impairment, dry eye, photophobia, cataract, conjunctivitis, diplopia, eye pain, visual field defect, asthenopia, eye hematoma, night blindness, periorbital edema, photosensitivity reaction, reduced visual acuity, vitreous floaters, blepharospasm, color blindness, eye edema, eye swelling, eyelid disorder, eyelid injury, eyelids pruritus, glaucoma, ophthalmic herpes zoster, and orbital edema

[a]Adverse events were reported by grouped terms or individual terms using the Medical Dictionary for Regulatory Activities, version 21.0.

Table S3. Patient and disease characteristics at baseline

Characteristic	All patients treated at the recommended dose (N = 472)
Age	
Median, years (range)	56.5 (18–93)
Distribution, n (%)	
≥ 18 to < 65 yr	352 (75)
≥ 65 yr	120 (25)
Female sex, n (%)	279 (59)
Ethnicity, n (%)	
Hispanic or Latino	14 (3)
Not Hispanic or Latino	445 (94)
Geographic region, n (%)	
United States	111 (24)
Asia	176 (37)
Other[a]	185 (39)
ECOG performance status, n (%)	
0	173 (37)
1	298 (63)
Stage 4 metastatic disease, n (%)	447 (95)
Gene fusion and tumor type, n (%)	
ROS1+ NSCLC[b]	335 (71)
NTRK+ solid tumor[c]	135 (29)
Other[d]	2 (< 1)

[a]Other regions included Australia, Canada, and Europe.

[b]The *ROS1*+ NSCLC cohort includes patients who were ROS1 TKI-naïve (n = 113) and patients with 1 prior ROS1 TKI and no prior chemotherapy or immunotherapy (n = 104; prior crizotinib, n = 81; prior entrectinib, n = 20).

[c]The *NTRK*+ solid tumor cohort includes patients who were TRK TKI-naïve (n = 53) and TRK TKI-pretreated (n = 82; prior larotrectinib, n = 48; prior entrectinib, n = 34).

[d]Other includes patients with *ROS1*+ non-NSCLC, *ALK*+ gene fusions, or any gene fusions with discordant results between local FISH test and central laboratory test.

Abbreviations: ECOG, Eastern Cooperative Oncology Group; FISH, fluorescent in situ hybridization; NSCLC, non-small cell lung cancer; TKI, tyrosine kinase inhibitor.

Table S4. Summary of AEs in all patients treated at the recommended dose in Asian and non-Asian patients

	TEAEs		TRAEs	
**Events, n (%)**	**Asian (n = 212)**	**Non-Asian (n = 260)**	**Asian (n = 212)**	**Non-Asian (n = 260)**
Events that led to dose interruption	122 (58)	139 (53)	93 (44)	89 (34)
Events that led to dose reduction	97 (46)	102 (39)	90 (42)	89 (34)
Events that led to treatment discontinuation	16 (8)	31 (12)	5 (2)	15 (6)
Serious events	75 (35)	111 (43)	22 (10)	23 (9)
Grade ≥ 3 events	113 (53)	156 (60)	75 (35)	75 (29)
Fatal events	13 (6)	15 (6)	1 (< 1)	1 (< 1)

Abbreviations: AE, adverse event; TEAE, treatment-emergent adverse event; TRAE, treatment-related adverse event.

Table S5. Summary of AEs in all patients treated at the recommended dose by age

	TEAEs			TRAEs		
**Events, n (%)**	**≥ 18 to < 65 years (n = 352)**	**≥ 65 to < 75 years (n = 89)**	**≥ 75 years (n = 31)**	**≥ 18 to < 65 years (n = 352)**	**≥ 65 to < 75 years (n = 89)**	**≥ 75 years (n = 31)**
Events that led to dose interruption	183 (52)	55 (62)	23 (74)	121 (34)	44 (49)	17 (55)
Events that led to dose reduction	128 (36)	50 (56)	21 (68)	114 (32)	47 (53)	18 (58)
Events that led to treatment discontinuation	26 (7)	14 (16)	7 (23)	8 (2)	8 (9)	4 (13)
Serious events	126 (36)	40 (45)	20 (65)	27 (8)	13 (15)	5 (16)
Grade ≥ 3 events	193 (55)	55 (62)	21 (68)	102 (29)	36 (40)	12 (39)
Fatal events	18 (5)	8 (9)	2 (6)	1 (< 1)	0	1 (3)

Abbreviations: AE, adverse event; TEAE, treatment-emergent adverse event; TRAE, treatment-related adverse event.

Table S6. Summary of AEs in all patients treated at the recommended dose in the *ROS1*+ NSCLC and *NTRK*+ solid tumor populations

	TEAEs		TRAEs	
Events, n (%)	*ROS1*+ NSCLC population (n = 335)	*NTRK*+ solid tumor population (n = 135)	*ROS1*+ NSCLC population (n = 335)	*NTRK*+ solid tumor population (n = 135)
Any event	333 (99)	134 (99)	320 (96)	131 (97)
Events that led to dose interruption	186 (56)	74 (55)	121 (36)	60 (44)
Events that led to dose reduction	134 (40)	64 (47)	116 (35)	62 (46)
Events that led to treatment discontinuation	35 (10)	12 (9)	15 (4)	5 (4)
Serious events	133 (40)	53 (39)	27 (8)	18 (13)
Grade ≥ 3 events	191 (57)	77 (57)	101 (30)	49 (36)
Fatal events	21 (6)	7 (5)	1 (< 1)	1 (1)

Abbreviations: AE, adverse event; NSCLC, non-small cell lung cancer; TEAE, treatment-emergent adverse events; TRAE, treatment-related adverse event.

Table S7. Summary of AEs in all patients treated at the recommended dose by prior treatment history

	***ROS1*+ NSCLC population (n = 335)**			***NTRK*+ solid tumor population (n = 135)**	
	**TKI-naïve (n = 113)**	**1 prior *ROS1* TKI and no prior chemotherapy or immunotherapy (n = 104)**	**TKI-pretreated (n = 198)**	**TKI-naïve (n = 53)**	**TKI-pretreated (n = 82)**
**TEAEs**					
Events that led to dose interruption	71 (63)	55 (53)	103 (52)	38 (72)	36 (44)
Events that led to dose reduction	61 (54)	39 (38)	65 (33)	34 (64)	30 (37)
Events that led to treatment discontinuation	16 (14)	11 (11)	18 (9)	7 (13)	5 (6)
Serious events	50 (44)	36 (35)	68 (34)	24 (45)	29 (35)
Grade ≥ 3 events	72 (64)	50 (48)	102 (52)	33 (62)	44 (54)

Fatal events	8 (7)	3 (3)	11 (6)	2 (4)	5 (6)
**TRAEs**					
Events that led to dose interruption	53 (47)	36 (35)	61 (31)	33 (62)	27 (33)
Events that led to dose reduction	55 (49)	31 (30)	55 (28)	34 (64)	28 (34)
Events that led to treatment discontinuation	9 (8)	4 (4)	6 (3)	3 (6)	2 (2)
Serious events	13 (12)	8 (8)	14 (7)	8 (15)	10 (12)
Grade ≥ 3 events	46 (41)	27 (26)	51 (26)	22 (42)	27 (33)
Fatal events	0	1 (1)	1 (1)	0	1 (1)

Abbreviations: AE, adverse event; NSCLC, non-small cell lung cancer; TEAE, treatment-emergent adverse event; TKI, tyrosine kinase inhibitor; TRAE, treatment-related adverse event.

Table S8. Summary of AEs in all patients treated at the recommended dose by prior TKI therapy

	ROS1+ NSCLC TKI-pretreated cohort (n = 104)		*NTRK*+ solid tumor TKI-pretreated cohort (n = 82)	
	**Prior crizotinib (n = 81)**	**Prior entrectinib (n = 20)**	**Prior entrectinib (n = 34)**	**Prior larotrectinib (n = 48)**
**TEAEs**				
Events that led to dose interruption	43 (53)	11 (55)	14 (41)	22 (46)
Events that led to dose reduction	30 (37)	8 (40)	12 (35)	17 (35)
Events that led to treatment discontinuation	9 (11)	2 (10)	2 (6)	3 (6)
Serious events	25 (31)	10 (50)	11 (32)	16 (33)
Grade ≥ 3 events	37 (46)	12 (60)	16 (47)	26 (54)
Fatal events	2 (2)	1 (5)	2 (6)	2 (4)
**TRAEs**				
Events that led to dose interruption	31 (38)	4 (20)	10 (29)	16 (33)

Events that led to dose reduction	26 (32)	4 (20)	11 (32)	16 (33)
Events that led to treatment discontinuation	4 (5)	0	1 (3)	1 (2)
Serious events	5 (6)	3 (15)	2 (6)	5 (10)
Grade ≥ 3 events	23 (28)	3 (15)	8 (24)	16 (33)
Fatal events	0	1 (5)	0	0

Abbreviations: AE, adverse event; TEAE, treatment-emergent adverse event; TKI, tyrosine kinase inhibitor; TRAE, treatment-related adverse event.

Table S9. Summary of reasons for patients in the safety population who did not dose increase to 160 mg BID

	***ROS1*+ NSCLC population (n = 335)**	***NTRK*+ solid tumor population (n = 135)**	**Total patients in the safety population (n = 472)[a]**
Patients with dose increased to 160 mg BID, n (%)	290 (86)	112 (83)	404 (86)
Patients without dose increased to 160 mg BID, n (%)	45 (13)	21 (16)	66 (14)[b]
Reasons for dose not increased to 160 mg BID, n (%)			
Patients treated with repotrectinib for < 14 days	0	2 (1)	2 (< 1)
Grade ≥ 3 TRAEs[c]	3 (1)	0	3 (1)
CNS AEs[d]	14 (4)	10 (7)	24 (5)
Grade ≥ 3 lab abnormalities[e]	6	0	6 (1)
Other AEs[f]	8	6	25 (5)
Other[g]	8	0	8 (2)
Investigator decision	3	5	8 (2)

[a]Included 2 other treated patients and both patients dose increased to 160 mg BID. Other treated patients may include patients with *ROS1*+ non-NSCLC, *ALK*+ gene fusions, and any gene fusions with discordant results between local FISH test and central laboratory test.

[b]Included 3 patients from the Midazolam substudy who did not dose increase to 160 mg BID because of grade 3 dyspnea, grade 2 thromboembolic event, grade 2 gait disturbance, grade 1 confusion, and grade 3 vertigo.

[c]Included muscular cramps at the sole of the feet, urinary tract infection, and anemia.

[d]Included ataxia, paresthesia, dizziness/lightheadedness, pleural effusion, and neurologic symptoms.

[e]Included increased blood creatine phosphokinase, increased alanine aminotransferase, hepatic toxicity, low neutrophile counts, and liver intolerance.

[f]Included dysphagia, arthralgia (wrist, ankles, shoulders, and knees), constipation, wheezing, anemia, vertigo, thrombocytosis, neuralgia, increased gamma-glutamyl transferase, cholelithiasis, dizziness, dysgeusia, memory impairment, disturbance in attention, somnolence, pneumonia, and abnormal liver function.

[g]Included death prior to cycle 1 day 15, disease progression prior to cycle 1 day 15, and patient's decision.

Abbreviations: AE, adverse event; BID, twice daily; CNS, central nervous system; FISH, fluorescent in situ hybridization; NSCLC, non-small cell lung cancer; TRAE, treatment-related adverse event.

Table S10. Incidence of dizziness and ataxia by individual terms in all patients treated at the recommended dose

	TEAEs		TRAEs	
**Events, n (%)**	**All grades**	**Grade ≥ 3**	**All grades**	**Grade ≥ 3**
Dizziness[a]	312 (66)	14 (3)	288 (61)	14 (3)
Dizziness	299 (63)	13 (3)	275 (58)	13 (3)
Vertigo	18 (4)	1 (< 1)	16 (3)	1 (< 1)
Dizziness postural	6 (1)	0	5 (1)	0
Dizziness exertional	1 (< 1)	0	0	0
Vertigo positional	1 (< 1)	0	0	0
Ataxia[a]	148 (31)	3 (1)	139 (29)	2 (< 1)
Ataxia	111 (24)	2 (< 1)	107 (23)	1 (< 1)
Gait disturbance	28 (6)	1 (< 1)	22 (5)	1 (< 1)
Balance disorder	19 (4)	0	17 (4)	0
Cerebellar ataxia	2 (< 1)	0	1 (< 1)	0
Coordination abnormal	1 (< 1)	0	1 (< 1)	0
Nystagmus	1 (< 1)	0	1 (< 1)	0
Dizziness events that led to dose interruption[a]	49 (10)[b]		46 (10)[c]	
Dizziness events that led to dose reduction[a]	59 (13)[d]		57 (12)[e]	

Dizziness events that led to treatment discontinuation[a]	0	0
Ataxia events that led to dose interruption[a]	27 (6)[f]	26 (6)[g]
Ataxia events that led to dose reduction[a]	41 (9)[h]	40 (8)[i]
Ataxia events that led to treatment discontinuation[a]	1 (< 1)[j]	1 (< 1)[k]

[a]Grouped term.

[b]Dose interruptions due to TEAEs by individual terms: dizziness (n = 47; 10%), dizziness postural (n = 1; < 1%), and vertigo (n = 1; < 1%).

[c]Dose interruptions due to TRAEs by individual terms: dizziness (n = 44; 9%), dizziness postural (n = 1; < 1%), and vertigo (n = 1; < 1%).

[d]Dose reductions due to TEAEs by individual terms: dizziness (n = 57; 12%) and vertigo (n = 3; 1%).

[e]Dose reductions due to TRAEs by individual terms: dizziness (n = 55; 12%) and vertigo (n = 3; 1%).

[f]Dose interruptions due to TEAEs by individual terms: ataxia (n = 20; 4%), gait disturbance (n = 3; 1%), balance disorder (n = 2; < 1%), cerebellar ataxia (n = 1; < 1 %), nystagmus (n = 1, < 1%).

[g]Dose interruptions due to TRAEs by individual terms: ataxia (n = 19; 4%), gait disturbance (n = 3; 1%), balance disorder (n = 2; < 1%), cerebellar ataxia (n = 1; < 1%), and nystagmus (n = 1; < 1%).

[h]Dose reductions due to TEAEs by individual terms: ataxia (n = 32; 7%), balance disorder (n = 4; 1%), gait disturbance (n = 4; 1 %), cerebellar ataxia (n = 1; < 1%).

[i]Dose reductions due to TRAEs by individual terms: ataxia (n = 31; 7%), balance disorder (n = 4; 1%), gait disturbance (n = 4; 1%), and cerebellar ataxia (n = 1; < 1%).

[j]Dose discontinuations due to TEAEs by individual terms: balance disorder (n = 1; < 1%).

[k]Dose discontinuations due to TRAEs by individual terms: balance disorder (n = 1; < 1%).

Abbreviations: TEAE, treatment-emergent adverse event; TRAE, treatment-related adverse event.

Table S11. Subsequent downgrading with resolution of TRAEs after dose modification in all patients treated at the recommended dose[a]

Events	Patients who reported TRAE, n	Patients with dose modification due to TRAE, n (%)[b]	Patients with subsequent downgrading or resolution of TRAE after dose modification, n (%)[c]	Patients with subsequent resolution of TRAE after dose modification, n (%)[c]
Dizziness	288	69 (24)	54 (78)	42 (61)
Ataxia	139	47 (34)	39 (83)	26 (55)
Hepatotoxicity	114	11 (10)	9 (82)	9 (82)
Cognitive impairment	89	12 (13)	9 (75)	6 (50)
Peripheral neuropathy	82	8 (10)	6 (75)	6 (75)
Muscular weakness	78	36 (46)	25 (69)	18 (50)
Myalgia	77	11 (14)	9 (82)	7 (64)
Pneumonitis	13	7 (54)	6 (86)	6 (86)

[a]Only includes TRAEs that were commonly managed with dose modification (> 10% of patients with AEs who had dose modification).

[b]Includes patients with dose reduction only, with dose interruption only, and with dose reduction and dose interruption.

[c]Based on patients with dose modification due to TRAE.

Abbreviations: AE, adverse event; TRAE, treatment-related adverse event.

Table S12. Summary of withdrawal pain in all patients treated at the recommended dose

	Treatment-emergent withdrawal pain		Treatment-related withdrawal pain	
	Any grade	Grade ≥ 3	Any grade	Grade ≥ 3
**Patients with treatment interruption for any reason, n (%)[a]**	39 (14)	2 (1)	17 (6)	0
Median time to onset of withdrawal pain, days (range)	2 (1–39)	1 (1–1)	2 (1–9)	-
Median time to resolution of withdrawal pain, days (95% CI)[b,c]	15 (8–20)	19.5 (3–NA)	16 (7–40)	-
Range[d]	1–1022+	3–36	3–1022+	-
**Patients who discontinued treatment for any reason, n (%)[e]**	36 (12)	7 (2)	15 (5)	5 (2)
Median time to onset of withdrawal pain, days (range)	5 (2–24)	3 (2–8)	3 (2–9)	3 (2–5)
Median time to resolution of withdrawal pain, days (95% CI)[b,c]	138 (33–NA)	14 (8–NA)	138 (8–NA)	14 (8–NA)
Range[d]	1–743+	8–259+	4–743+	8–259+

[a]Percentages based on total number of patients who discontinued treatment for any reason (n = 286).

[b]Events without a stop date or with a stop date equal to the death as well as grade 5 events are considered unresolved.

[c]From Kaplan-Meier estimation.

[d]Symbol + indicates a censored value.

[e]Percentages based on total number of patients who discontinued treatment for any reason (n = 311).

Abbreviations: CI, confidence interval; NA, not applicable.

Table S13. Incidence of dysgeusia by individual terms in all patients treated at the recommended dose

	TEAEs		TRAEs	
**Events, n (%)**	**All grades**	**Grade ≥ 3**	**All grades**	**Grade ≥ 3**
Dysgeusia	272 (58)	0	257 (54)	0
Dysgeusia	250 (53)	0	237 (50)	0
Taste disorder	17 (4)	0	16 (3)	0
Ageusia	5 (1)	0	4 (1)	0
Sensory disturbance	3 (1)	0	2 (< 1)	0
Allodynia	1 (< 1)	0	1 (< 1)	0
Hypogeusia	1 (< 1)	0	1 (< 1)	0
Sensory loss	1 (< 1)	0	1 (< 1)	0
Events that led to dose interruption	3 (1)[a]		3 (1)[b]	
Events that led to dose reduction	2 (< 1)[c]		2 (< 1)[d]	
Events that led to treatment discontinuation	0		0	

[a]Dose interruption due to TEAEs by individual terms: dysgeusia (n = 3; 1%).

[b]Dose interruption due to TRAEs by individual terms: dysgeusia (n = 3; 1%).

[c]Dose reductions due to TEAEs by individual terms: dysgeusia (n = 2; < 1%).

[d]Dose reductions due to TRAEs by individual terms: dysgeusia (n = 2; < 1%).

Abbreviations: TEAE, treatment-emergent adverse event; TRAE, treatment-related adverse event.

Table S14. Incidence of peripheral neuropathy by individual terms in all patients treated at the recommended dose

	TEAEs		TRAEs	
**Events, n (%)**	**All grades**	**Grade ≥ 3**	**All grades**	**Grade ≥ 3**
Peripheral neuropathy	99 (21)	5 (1)	82 (17)	5 (1)
Neuralgia	63 (13)	2 (< 1)	51 (11)	2 (< 1)
Peripheral sensory neuropathy	22 (5)	1 (< 1)	20 (4)	1 (< 1)
Neuropathy peripheral	19 (4)	1 (< 1)	16 (3)	1 (< 1)
Peripheral motor neuropathy	5 (1)	1 (< 1)	2 (< 1)	1 (< 1)
Polyneuropathy	1 (< 1)	0	1 (< 1)	0
Events that led to dose interruption	7 (1)[a]		6 (1)[b]	
Events that led to dose reduction	6 (1)[c]		4 (1)[d]	
Events that led to treatment discontinuation	1 (< 1)[e]		1 (< 1)[f]	

[a]Dose interruption due to TEAEs by individual terms: neuropathy peripheral (n = 3; 1%), neuralgia (n = 2; < 1%), and peripheral sensory neuropathy (n = 2; < 1%).

[b]Dose interruption due to TRAEs by individual terms: neuropathy peripheral (n = 2; < 1%), neuralgia (n = 2; < 1%), and peripheral sensory neuropathy (n = 2; < 1%).

[c]Dose reductions due to TEAEs by individual terms: neuropathy peripheral (n = 3; 1%), neuralgia (n = 2; < 1%), and peripheral sensory neuropathy (n = 1, < 1%).

[d]Dose reductions due to TRAEs by individual terms: neuropathy peripheral (n = 2; < 1%), neuralgia (n = 1; < 1%), and peripheral sensory neuropathy (n = 1; < 1%).

[e]Treatment discontinuation due to TEAEs by individual terms: peripheral motor neuropathy (n = 1; < 1%).

[f]Treatment discontinuation due to TRAEs by individual terms: peripheral motor neuropathy (n = 1; < 1%).

Abbreviations: TEAE, treatment-emergent adverse event; TRAE, treatment-related adverse event.

Table S15. Incidence of paresthesia by individual terms in all patients treated at the recommended dose

	TEAEs		TRAEs	
**Events, n (%)**	**All grades**	**Grade ≥ 3**	**All grades**	**Grade ≥ 3**
Paresthesia	190 (40)	4 (1)	166 (35)	4 (1)
Paresthesia	165 (35)	3 (1)	144 (31)	3 (1)
Hypoesthesia	18 (4)	1 (< 1)	14 (3)	1 (< 1)
Hyperesthesia	13 (3)	0	13 (3)	0
Dysesthesia	9 (2)	0	7 (1)	0
Burning sensation	5 (1)	0	5 (1)	0
Anesthesia	1 (< 1)	0	1 (< 1)	0
Formication	1 (< 1)	0	1 (< 1)	0
Events that led to dose interruption	8 (2)[a]		6 (1)[b]	
Events that led to dose reduction	13 (3)[c]		13 (3)[d]	
Events that led to treatment discontinuation	0		0	

[a]Dose interruption due to TEAEs by individual terms: paresthesia (n = 6; 1%), hyperesthesia (n = 1; < 1%), and hypoesthesia (n = 1; < 1%).

[b]Dose interruption due to TRAEs by individual terms: paresthesia (n = 5; 1%) and hyperesthesia (n = 1; < 1%).

[c]Dose reductions due to TEAEs by individual terms: paresthesia (n = 9; 2%), dysesthesia (n = 2; < 1%), hyperesthesia (n = 1; < 1%), and hypoesthesia (n = 1; < 1%).

[d]Dose reductions due to TRAEs by individual terms: paresthesia (n = 9; 2%), dysesthesia (n = 2; < 1%), hyperesthesia (n = 1; < 1%), and hypoesthesia (n = 1; < 1%).

Abbreviations: TEAE, treatment-emergent adverse event; TRAE, treatment-related adverse event.

Table S16. Incidence of cognitive impairment by individual terms in all patients treated at the recommended dose

	TEAEs		TRAEs	
**Events, n (%)**	**All grades**	**Grade ≥ 3**	**All grades**	**Grade ≥ 3**
Cognitive impairment	115 (24)	7 (1)	89 (19)	3 (1)
Memory impairment	69 (15)	1 (< 1)	56 (12)	1 (< 1)
Disturbance in attention	58 (12)	0	46 (10)	0
Cognitive disorder	33 (7)	0	29 (6)	0
Confusional state	8 (2)	1 (< 1)	4 (1)	0
Delirium	5 (1)	0	0	0
Amnesia	4 (1)	0	3 (1)	0
Attention deficit hyperactivity disorder	4 (1)	0	4 (1)	0
Aphasia	3 (1)	0	2 (< 1)	0
Altered state of consciousness	2 (< 1)	1 (< 1)	0	0
Depressed level of consciousness	2 (< 1)	2 (< 1)	2 (< 1)	2 (< 1)
Neurological decompensation	2 (< 1)	2 (< 1)	0	0
Bradyphrenia	1 (< 1)	0	1 (< 1)	0
Delusion	1 (< 1)	0	1 (< 1)	0
Dysgraphia	1 (< 1)	0	0	0

Hallucination	1 (< 1)	0	1 (< 1)	0
Mental status changes	1 (< 1)	0	1 (< 1)	0
Events that led to dose interruption	9 (2)[a]		7 (1)[b]	
Events that led to dose reduction	11 (2)[c]		11 (2)[d]	
Events that led to treatment discontinuation	3 (1)[e]		0	

[a]Dose interruptions due to TEAEs by individual terms: confusional state (n = 3; 1%), depressed level of consciousness (n = 2; < 1%), disturbance in attention (n = 2; < 1%), memory impairment (n = 2; < 1%), delirium (n = 1; < 1%), delusion (n = 1; < 1%), and mental status changes (n = 1; < 1%).

[b]Dose interruptions due to TRAEs by individual terms: depressed level of consciousness (n = 2; < 1%), disturbance in attention (n = 2; < 1%), memory impairment (n = 2; < 1%), confusional state (n = 1; < 1%), delusion (n = 1; < 1%), and mental status changes (n = 1; < 1%).

[c]Dose reductions due to TEAEs by individual terms: disturbance in attention (n = 3; 1%), memory impairment (n = 3; 1%), confusional state (n = 2; < 1%), depressed level of consciousness (n = 2; < 1%), attention deficit hyperactivity disorder (n = 1; < 1%), cognitive disorder (n = 1; < 1%), mental status changes (n = 1; < 1%).

[d]Dose reductions due to TRAEs by individual terms: disturbance in attention (n = 3; 1%), memory impairment (n = 3; 1%), confusional state (n = 2; < 1%), depressed level of consciousness (n = 2; < 1%), attention deficit hyperactivity disorder (n = 1; < 1%), cognitive disorder (n = 1; < 1%), mental status changes (n = 1; < 1%).

[e]Treatment discontinuation due to TEAEs by individual terms: neurological decompensation (n = 2; < 1%) and depressed level of consciousness (n = 1; < 1%).

Abbreviations: TEAE, treatment-emergent adverse event; TRAE, treatment-related adverse event.

Table S17. Incidence of pneumonitis by individual terms in all patients treated at the recommended dose

	TEAEs		TRAEs	
**Events, n (%)**	**All grades**	**Grade ≥ 3**	**All grades**	**Grade ≥ 3**
Pneumonitis	16 (3)[a]	5 (1)	13 (3)	4 (1)
Pneumonitis	15 (3)	5 (1)	12 (3)	4 (1)
Interstitial lung disease	1 (< 1)	0	1 (< 1)	0
Events that led to dose interruption	8 (2)[b]		6 (1)[c]	
Events that led to dose reduction	3 (1)[d]		3 (1)[e]	
Events that led to treatment discontinuation	5 (1)[f]		5 (1)[g]	

[a]One patient reported radiation pneumonitis after receiving radiotherapy to treat disease progression while on study, with intent to treat beyond progression.

[b]Dose interruptions due to TEAEs by individual terms: pneumonitis (n = 8; 2%).

[c]Dose interruptions due to TRAEs by individual terms: pneumonitis (n = 6; 1%).

[d]Dose reductions due to TEAEs by individual terms: pneumonitis (n = 3; 1%).

[e]Dose reductions due to TRAEs by individual terms: pneumonitis (n = 3; 1%).

[f]Treatment discontinuation due to TEAEs by individual terms: pneumonitis (n = 5; 1%).

[g]Treatment discontinuation due to TRAEs by individual terms: pneumonitis (n = 5; 1%).

Abbreviations: TEAE, treatment-emergent adverse event; TRAE, treatment-related adverse event.

Table S18. Incidence of fractures by individual terms in all patients treated at the recommended dose

	TEAEs		TRAEs	
**Events, n (%)**	**All grades**	**Grade ≥ 3**	**All grades**	**Grade ≥ 3**
Fractures	15 (3)	2 (< 1)	1 (< 1)	0
Foot fracture	3 (1)	0	1 (< 1)	0
Rib fracture	2 (< 1)	0	0	0
Spinal compression fracture	2 (< 1)	1 (< 1)	0	0
Acetabulum fracture	1 (< 1)	0	0	0
Ankle fracture	1 (< 1)	0	0	0
Femur fracture	1 (< 1)	1 (< 1)	0	0
Fibula fracture	1 (< 1)	0	0	0
Forearm fracture	1 (< 1)	0	0	0
Fracture	1 (< 1)	0	0	0
Sternal fracture	1 (< 1)	0	0	0
Upper limb fracture	1 (< 1)	0	0	0
Events that led to dose interruption	3 (1)[a]		0	
Events that led to dose reduction	0		0	
Events that led to treatment discontinuation	1 (< 1)[b]		0	

[a]Dose interruptions due to TEAEs by individual terms: femur fracture (n = 1; < 1%), rib fracture (n = 1; < 1%), spinal compression fracture (n = 1; < 1%).

[b]Treatment discontinuation due to TEAEs by individual terms: femur fracture (n = 1; < 1%).

Abbreviations: TEAE, treatment-emergent adverse event; TRAE, treatment-related adverse event.

Table S19. Incidence of vision disorders by individual terms in all patients treated at the recommended dose

	TEAEs		TRAEs	
**Events, n (%)**	**All grades**	**Grade ≥ 3**	**All grades**	**Grade ≥ 3**
Vision disorders	67 (14)	3 (1)	31 (7)	1 (< 1)
Blurred vision	19 (4)	0	9 (2)	0
Visual impairment	10 (2)	1 (< 1)	2 (< 1)	0
Dry eye	8 (2)	0	4 (1)	0
Photophobia	5 (1)	0	2 (< 1)	0
Cataract	3 (1)	0	0	0
Conjunctivitis	3 (1)	0	0	0
Diplopia	3 (1)	0	1 (< 1)	0
Eye pain	3 (1)	0	1 (< 1)	0
Visual field defect	3 (1)	0	1 (< 1)	0
Asthenopia	2 (< 1)	0	1 (< 1)	0
Eye hematoma	2 (< 1)	0	0	0
Night blindness	2 (< 1)	0	1 (< 1)	0
Periorbital edema	2 (< 1)	1 (< 1)	2 (< 1)	1 (< 1)
Photosensitivity reaction	2 (< 1)	0	2 (< 1)	0

Reduced visual acuity	2 (< 1)	0	0	0
Vitreous floaters	2 (< 1)	0	0	0
Blepharospasm	1 (< 1)	0	1 (< 1)	0
Color blindness	1 (< 1)	0	1 (< 1)	0
Eye edema	1 (< 1)	0	1 (< 1)	0
Eye swelling	1 (< 1)	0	1 (< 1)	0
Eyelid disorder	1 (< 1)	0	0	0
Eyelid injury	1 (< 1)	0	0	0
Eyelid pruritus	1 (< 1)	0	1 (< 1)	0
Glaucoma	1 (< 1)	0	0	0
Ophthalmic herpes zoster	1 (< 1)	1 (< 1)	0	0
Orbital edema	1 (< 1)	0	1 (< 1)	0
Events that led to dose interruption	7 (1)[a]		3 (1)[b]	
Events that led to dose reduction	1 (< 1)[c]		1 (< 1)[d]	
Events that led to treatment discontinuation	1 (< 1)[e]		1 (< 1)[f]	

[a]Dose interruptions due to TEAEs by individual terms: blurred vision (n = 2; < 1%), cataract (n = 1; < 1%), color blindness (n = 1; < 1%), eyelid injury (n = 1; < 1%), ophthalmic herpes zoster (n = 1; < 1%), periorbital edema (n = 1; < 1%), and visual impairment (n = 1; < 1%).

[b]Dose interruptions due to TRAEs by individual terms: blurred vision (n = 2; < 1%), color blindness (n = 1; < 1%), and periorbital edema (n = 1; < 1%). [c]Dose reduction due to TEAEs by individual terms: color blindness (n = 1; < 1%).

[d]Dose reduction due to TRAEs by individual terms: color blindness (n = 1; < 1%).

[e]Treatment discontinuation due to TEAEs by individual terms: color blindness (n = 1; < 1%).

[f]Treatment discontinuation due to TRAEs by individual terms: color blindness (n = 1; < 1%).

Abbreviations: TEAE, treatment-emergent adverse event; TRAE, treatment-related adverse event.

REFERENCES

1. U.S. Department of Health and Human Services. Common Terminology Criteria for Adverse Events (CTCAE), Version 5.0. November 27, 2017. Accessed May 20, 2024. https://ctep.cancer.gov/protocolDevelopment/electronic_applications/docs/CTCAE_v5_Quick_Reference_5x7.pdf.

2. Shim A, Trone D, Reynolds M, et al. PCR158 Patient reported outcomes (PRO) from ongoing phase 2 registrational trial of repotrectinib in patients with ROS1-positive advanced or metastatic non-small cell lung cancer (TRIDENT-1). *Value Health* 2022;25(supplement):S421.

3. Sherif B, Besse B, Solomon B, et al. PCR181 Health-related quality of life of patients treated with repotrectinib for neurotrophic tyrosine receptor kinase (*NTRK*)–positive advanced solid tumors: results from TRIDENT-1. *Value Health* 2023;26(supplement):S484.

4. Besse B, Lin JJ, Bazhenova L, et al. Repotrectinib in *NTRK* fusion–positive advanced solid tumors: a phase 1/2 trial. *Nat Med.* 2026;32:682–689.
